# Hypoxia-Mediated Complement 1q Binding Protein Regulates Metastasis and Chemoresistance in Triple-Negative Breast Cancer and Modulates the PKC-NF-κB-VCAM-1 Signaling Pathway

**DOI:** 10.3389/fcell.2021.607142

**Published:** 2021-02-23

**Authors:** Hao Wu, Yijun Chu, Shanshan Sun, Guozheng Li, Shouping Xu, Xianyu Zhang, Yongdong Jiang, Song Gao, Qin Wang, Jian Zhang, Da Pang

**Affiliations:** ^1^Sino-Russian Medical Research Center, Harbin Medical University Cancer Hospital, Harbin, China; ^2^Translational Medicine Research and Cooperation Center of Northern China, Harbin Medical University, Harbin, China; ^3^Heilongjiang Academy of Medical Sciences, Harbin, China; ^4^Department of Breast Surgery, Harbin Medical University Cancer Hospital, Harbin, China

**Keywords:** hypoxia, TNBC, C1QBP, metastasis, chemoresistance

## Abstract

**Objectives:**

Complement 1q binding protein (C1QBP/HABP1/p32/gC1qR) has been found to be overexpressed in triple-negative breast cancer (TNBC). However, the underlying mechanisms of high C1QBP expression and its role in TNBC remain largely unclear. Hypoxia is a tumor-associated microenvironment that promotes metastasis and paclitaxel (PTX) chemoresistance in tumor cells. In this study, we aimed to assess C1QBP expression and explore its role in hypoxia-related metastasis and chemoresistance in TNBC.

**Materials and Methods:**

RNA-sequencing of TNBC cells under hypoxia was performed to identify C1QBP. The effect of hypoxia inducible factor 1 subunit alpha (HIF-1α) on C1QBP expression was investigated using chromatin immunoprecipitation (ChIP) assay. The role of C1QBP in mediating metastasis, chemoresistance to PTX, and regulation of metastasis-linked vascular cell adhesion molecule 1 (VCAM-1) expression were studied using *in vitro* and *in vivo* experiments. Clinical tissue microarrays were used to verify the correlation of C1QBP with the expression of HIF-1α, VCAM-1, and RELA proto-oncogene nuclear factor-kappa B subunit (P65).

**Results:**

We found that hypoxia-induced HIF-1α upregulated C1QBP. The inhibition of C1QBP notably blocked metastasis of TNBC cells and increased their sensitivity to PTX under hypoxic conditions. Depletion of C1QBP decreased VCAM-1 expression by reducing the amount of P65 in the nucleus and suppressed the activation of hypoxia-induced protein kinase C-nuclear factor-kappa B (PKC-NF-κB) signaling.

immunohistochemistry (IHC) staining of the tissue microarray showed positive correlations between the C1QBP level and those of HIF-1α, P65, and VCAM-1.

**Conclusion:**

Targeting C1QBP along with PTX treatment might be a potential treatment for TNBC patients.

## Graphical Abstract

In TNBC cells, the inhibition of C1QBP suppresses PKC–NF-κB signaling in cytoplasm, upregulating Iκβα expression, thereby decreasing the VCAM-1 expression by lowering p65 level in nucleus in normoxic conditions. When TNBC cells are exposed to hypoxic conditions, HIF-1α upregulates C1QBP expression. The inhibition of C1QBP notably suppresses the activation of hypoxia-induced PKC–NF-κB signaling and decreases VCAM-1 level, resulting in metastasis blocking and PTX drug chemoresistance decreasing of TNBC cells.

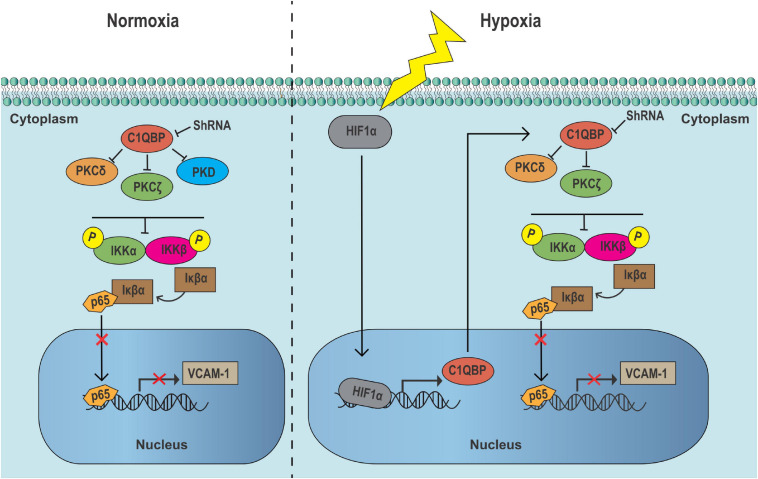

## Introduction

Based on data from the Global Cancer Statistics, breast cancer is one of most commonly diagnosed cancers (comprising 24.2% of cancers in women) and a major cause of death in women (15% of total mortalities); the incidence of breast cancer is continuously increasing, with 18 million people worldwide currently affected each year (Bray et al., 2018; Cruz et al., 2019).

Triple-negative breast cancer (TNBC) is a subtype that accounts for 15–20% of all breast cancer cases (Sharma, 2018). Patients with this subtype lack the estrogen receptor, progesterone receptor, and amplified expression of human epidermal growth factor receptor 2, indicating that no conventional targeted or endocrine therapy is available and chemotherapeutic agents are the only viable options for treating this disease (Cai et al., 2019; Yu et al., 2019). The overall survival of TNBC patients is shorter than that of patients suffering from other subtypes of breast cancer (Khan et al., 2019). Metastasis and resistance to chemotherapy are critical factors that reduce the survival of patients with TNBC (Jyotsana et al., 2019; Khan et al., 2019). TNBC cells can easily migrate from the invasive front to distant sites via capillaries and become engrafted into new microenvironments (Clevers and Nusse, 2012). Paclitaxel (PTX) is currently the most common chemotherapeutic agent for treating metastatic or non-metastatic breast cancer, especially for TNBC (Liu et al., 2017; Yan et al., 2018). Although TNBC cells are more sensitive to chemotherapy (Shetti et al., 2019), they usually metastasize and develop chemoresistance after a period of PTX treatment (Liu et al., 2017).

Intratumoral hypoxia is a common and characteristic microenvironment found in advanced cancers (Xiang et al., 2014). The average pressure of oxygen in primary breast cancers is 10 mm Hg, equivalent to 1.4% (v/v) O_2_; in contrast, the oxygen pressure in normal breast tissue is 65 mm Hg [9.3% (v/v) O_2_] (Vaupel et al., 2007; Zhang et al., 2016). Hypoxia-inducible factors (HIFs) are a family of essential master transcription factors that stimulate changes in gene expression in response to hypoxia (Semenza, 2012). It has been unequivocally established that HIFs promote metastasis (Xiang et al., 2014), PTX chemoresistance (Yan et al., 2018), and the expansion of breast cancer stem-like cells (Lu et al., 2015; Zhang et al., 2016) in TNBC. Thus, it is imperative to develop new therapeutic targets to reduce tumor metastasis and strengthen the combined treatment for hypoxia-induced sensitivity to TNBC chemotherapy.

Complement 1q binding protein (C1QBP) belongs to the hyaluronic acid family, and the mature protein, comprising 209 amino acids, resides in the cytoplasm, mitochondria, and membrane of mammalian cells. It is an important evolutionarily conserved multifunctional protein that is involved in multiple biological processes and specifically binds to the extracellular matrix (Kim et al., 2011; Feichtinger et al., 2017). An increasing number of studies have shown that this protein is closely associated with the development of malignant tumors (Saha et al., 2014; Li et al., 2017; Shi et al., 2017; Xie et al., 2019). We have previously reported high levels of C1QBP in breast cancer tissues compared to those in normal breast tissues (Chen et al., 2009); C1QBP overexpression was correlated with enhanced tumor metastasis to the lymph nodes of TNBC patients, which can serve as an independent prognostic indicator (Wang et al., 2015); silencing C1QBP suppresses the ability of TNBC cells to metastasize and inhibits their proliferation both *in vitro* and *in vivo*, suggesting that C1QBP promotes the progression of cancer in patients with TNBC (Niu et al., 2015).

Fogal et al., 2008 first observe that C1QBP, which acts as a mitochondrial surface protein, is primarily localized in hypoxic/nutrient-deprived regions within tumors (Fogal et al., 2008). Moreover, they further demonstrate that C1QBP is capable of promoting viability and tumorigenicity of breast cancer cells by regulating oxidative phosphorylation and opposing the shift of cell metabolism toward glycolysis, highly indicating that C1QBP may serve as a promising target for diagnosis and therapy for breast cancer (Fogal et al., 2010). Therefore, based on their important findings, the reasons behind the overexpression of C1QBP under hypoxia and its role in the tumorigenicity of TNBC need to be elucidated.

In this report, we showed that hypoxia induced the overexpression of C1QBP. Depletion of C1QBP downregulated the expression of vascular cell adhesion molecule 1 (VCAM-1) via the NF-κB signaling cascade. Moreover, we delineated novel functions of C1QBP in the migration, invasion, and drug resistance of TNBC cells. Further analysis of tissue specimens confirmed the correlation between the levels of C1QBP, hypoxia inducible factor 1 subunit alpha (HIF-1α), VCAM-1, and RELA proto-oncogene nuclear factor-kappa B subunit (P65) in TNBC patients, suggesting targeting C1QBP in the presence of PTX is a potential avenue for advanced therapy against TNBC.

## Materials and Methods

### Patient and Tissue Samples

All clinical samples in this study were collected from patients who had undergone surgery at the Harbin Medical University Cancer Hospital between January 2007 and December 2007. Prior to the survey, none of the patients had received radiation or chemotherapy. Tissue microarrays comprising 271 primary breast carcinoma tissues were performed. The Medical Ethics Committee of Harbin Medical University Cancer Hospital approved this study and all relevant information was provided to the participants (the ethics certification numbers: KY2016-34). The clinical pathological features of the patients are shown in Tables 1, 2.

**TABLE 1:** Distribution of selected variables in breast cancer cases.

**Variables**	**Cases, *n* = 271**
Age (year)	49.28
Age at menarche (year)	15.24
Age at first live birth (year)	25.66
Age at menopause (year)	49.32
**Menopausal status**	
Pre-menopausal	114 (42.07%)
Post-menopausal	157 (57.93%)
Breastfeeding duration (months)	24.80

*Data presented as the mean values or number (% of total number).*

**TABLE 2:** Summary of the clinicopathologic features of breast cancer studied.

**Variables**	**Number**	**Ratio (%)**
**Clinic stage (UICC)**		
0	0	0
I	52	19.19
II	179	66.05
III-IV	40	14.76
**Tumor size (cm)**		
≤2	115	42.44
>2	156	57.56
**LN involvement**		
Negative	142	52.40
Positive	129	47.60
**ER**		
Negative	96	35.42
Positive	175	64.58
**PR**		
Negative	54	19.93
Positive	217	80.07
**HER-2**		
Negative	181	66.79
Positive	90	33.21
**Ki67**		
Negative	14	5.17
Positive	257	94.83
**P53**		
Negative	189	69.74
Positive	82	30.26

### Immunohistochemistry

Tissue microarrays were incubated at 55°C overnight and then treated with dewaxed solution using concentration gradients of xylene and alcohol. After washing with pure water, microarrays were treated with 0.01 mol/L ethylene diamine tetraacetic acid (EDTA, pH 8.0) or citrate buffer (pH 6.0), and were then exposed to heat-induced epitope retrieval (100°C) for 5 min. After treatment with 3% H_2_O_2_ for 30 min, the slides were incubated with primary antibodies against C1QBP (BOSTER, catalog No. BM5284, 1:50 dilution), HIF-1α (Abcam, ab1, 1:50 dilution), P65 (BOSTER, catalog No. BA0610, 1:50 dilution), VCAM-1 (Abcam, ab134047, 1:50 dilution), and ki67 (Bioss, bs-233104R, 1:100 dilution) overnight at 4°C. The tissue microarrays were subsequently incubated with secondary antibodies (ZSGB-BIO, Goat anti-Rabbit IgG, PV-6001; ZSGB-BIO, Goat anti-Mouse: PV-6002) 28°C for 1 h. After washing with phosphate-buffered saline (PBS), target proteins were stained using 3,3′-diaminobenzidine (ZSGB-BIO: ZLI-9018 and K152317J) and counterstained with hematoxylin. Based on the intensity and degree of staining, the sections were analyzed and scored by two independent pathologists (double-blind). The final score was the product of the staining intensity scores (0: negative; 1: weak; 2: moderate; 3: strong) and staining area scores (0: 0–5%; 1: 6–25%; 2: 26–50%; 3: 51–75%; and 4: above 76%). Final score less than or equal to 4 was considered as low expression. Final score greater than or equal to 6 was considered as high expression (Niu et al., 2015; Lei et al., 2020).

### Cell Culture

We cultured Hs578T and EA.hy926 cells in Dulbecco’s modified Eagle medium (DMEM). Human umbilical vein endothelial cells (HUVECs) were cultured in Endothelial Cell Medium (ScienCell, Catalog No.: 1001). MDA-MB-468 cells were cultured in Roswell Park Medium Institute (RPMI)-1640 medium. All media were supplemented with 10% (v/v) fetal bovine serum (FBS) and 1% (v/v) penicillin/streptomycin. The cultures were incubated in a 5% CO_2_ and 95% air incubator [20% (v/v) O_2_]. A modular incubator chamber (Invivo2 1000) provided hypoxic conditions for the experiments (cells were placed in a mixture of 1% O_2_, 5% CO_2_, and 94% N_2_). All cell lines were verified using STR detection.

### Generation of C1QBP-Knockdown, HIF-1α-Knockdown, and ATP Binding Cassette Subfamily B Member 1-Overexpression Cell Lines

Synthetic small interfering RNAs (siRNAs) specific to C1QBP and HIF-1α mRNAs were designed and synthesized (GenePharma, Shanghai, China). Cells were transfected with siRNA duplexes using INTERFERin (Polyplus, 409-10) as per the kit instructions. The sequences of the siRNAs against C1QBP were as follows: si1 (5′–3′: CCUUGUGUUGGACUGUCAUTT, AUGACAGUCCAACACAAGGTT), si2 (5′–3′: CCACCUAAUG GAUUUCCUUTT, AAGGAAAUCCAUUAGGUGGTT), and si3 (5′–3′: GGAGCACCAGGAGUACAUUTT, AAUGUACUCCU GGUGCUCCTT). The sequences of the siRNAs against HIF-1α were (5′–3′): GCCGAGGAAGAACUAUGAATT and UUCAUAGUUCUUCCUCGGCTT. The HIF-1α inhibitor was purchased from MCE (2-Methoxyestradiol Cat. No. HY-12033, Shanghai, China). Lentiviral vectors for the shRNA against C1QBP and HIF-1α were obtained from OBiO (Shanghai, China) based on the siRNA sequences provided above. After infecting the cells with the lentiviral vectors containing the shRNAs, puromycin (0.5 mg/ml) was added to each transduced cell to enable the selection of the lentiviral vector, according to the kit protocol. ATP binding cassette subfamily B member 1 (MDR1)-overexpressed plasmid was synthesized (Genechem, Shanghai, China). MDA-MB-468 cells were transfected with 10ug MDR1-OE plasmid duplexes using jetPRIME (Polyplus, PT-114-15) as per the kit instructions.

### Quantitative Real-Time PCR

TRIzol reagent (Invitrogen, No. 15596-026) was used to extract total RNA, which was then transcribed into cDNA using the Transcriptor cDNA Synthesis kit (Roche; No. 4897030001). The mRNA levels were determined using real-time PCR with the SYBR Green Master kit (Roche; No. 4193914001). The cycling conditions were 95°C for 10 min followed by 40 cycles of 95°C for 15s and 60°C for 35s. The cycle threshold (CT) of the target gene was normalized using the β-actin mRNA levels using the 2^–ΔΔCt^ method with minor revisions, which provided the relative gene expression (Xu et al., 2019). The sequences of the primers used were as follows:

**Table:** 

**Genes (reference sequence)**	**Sequences**
C1QBP (NM.001212.4)	F: 5′-AACAACAGCATCCCACCAAC-3′
	R: 5′-TCACTCTCAGCCTCGTCTTC-3′
HIF-1α (NM_001530.4)	F: 5′-TTAGAAAGCAGTTCCGCAAGC-3′
	R: 5′-AGTGGTCATTAGCAGTAGG-3′
VCAM-1 (NM_001078.4)	F: 5′-TAGAGTTTTTGGAGGATACGGAT-3′
	R: 5′-ACATTGACATAAAGTGTTTGCGT-3′
ITGA4 (NM_000885.6)	F: 5′-AGCCCTAATGGAGAACCTTGT-3′
	R: 5′-CCAGTGGGGAGCTTATTTTCAT-3′
ITGB7 (NM_000889.3)	F: 5′-CCATTCAGCTTTCACCATGTGC-3′
	R: 5′-ACCTTCAGGCGAGTCCAGATT-3′
ITGB1 (NM_002211.4)	F: 5′-CCTACTTCTGCACGATGTGATG-3′
	R: 5′-CCTTTGCTACGGTTGGTTACATT-3′

**Table:** 

**Genes (reference sequence)**	**Sequences**
TNF (NM_000594.4)	F:5′-ACTTTGGAGTGATCGGCCCC-3′
	R: 5′-TTCTGTGTGCCAGACACCCTA-3′
β-actin (NM_001101.5)	F: 5′-TCGTGCGTGACATTAAGGAGAAG-3′
	R: 5′-GTTGAAGGTAGTTTCGTGGATGC-3′

### Western Blot

The cells were lysed in RIPA buffer (Beyotime Biotechnology, P0013B) containing protease inhibitor (Beyotime Biotechnology, P1045) for 30 min at 4°C. The lysates were clarified by centrifugation at 14,000 × *g* for 15 min at 4°C. Protein concentrations were determined using the BCA protein assay kit (Beyotime Biotechnology, P0012). Total proteins from each sample were separated using a precast 10% polyacrylamide-sodium dodecyl sulfate gel followed by blotting on polyvinylidene fluoride membranes. The membranes were blocked using 5% skim milk at 28°C for 1 h followed by overnight incubation with primary antibody at 4°C to detect the target protein. Primary antibodies included C1QBP (Abcam, ab24733, 1:1,000 dilution), HIF-1α (Novus, NB100-105, 1:500 dilution), VCAM-1 (CST, #13662, 1:1,000 dilution), the protein kinase C (PKC) isoform antibody sampler kit (CST, #9960, 1:1,000 dilution), and NF-κB pathway sampler kit (CST, #9936, 1:1,000 dilution). The membranes were incubated with anti-rabbit or anti-mouse secondary antibodies at room temperature for 1 h after washing with PBS containing Tween 20. The bands were observed using BeyoECL Star (Beyotime Biotechnology, P0018AS). Each protein was normalized to β-actin (Absin, abs132964, 1:1,000 dilution) or β-tubulin (SantaCruz, KM9002T, 1:1,000 dilution) levels. Cytoplasmic and nuclear protein extraction was performed using the Minute Cytoplasmic and Nuclear Extraction Kit (No. SC-003) following manufacturer’s instructions; the nuclear protein samples were normalized to the LaminB protein levels (Absin, abs131244).

### Transwell Assay

The transwell assay was performed in a 24-well plate to detect migration and invasion (Costar 3422, Corning, NY, United States). A polycarbonate membrane (8 μm pore size) separated the upper and lower compartments. In the upper chamber, cells (Hs578T: 5 × 10^4^ cells and MDA-MB-468: 1 × 10^5^ cells) were resuspended in DMEM or RPMI medium containing free FBS. DMEM or RPMI containing 20% FBS was added to the lower chamber. For invasion analysis, chamber inserts were included containing 200 mg/ml matrigel, and dried under sterile conditions, after which, cells were plated in the top chamber. Mitomycin C was added as 0.5 μg/ml (M5353, Sigma, United States). Following 48 h of incubation at 37°C, the membrane was rinsed using a PBS-soaked cotton swab and the lower side of the membrane was immobilized using 4% formaldehyde for 1 h; staining was performed overnight using crystal violet and the number of stained cells was counted under a light microscope.

### Cell Viability Assay

MDA-MB-468 (3.0 × 10^3^ cells per well) or Hs578T cells (1.0 × 10^3^ cells per well) were seeded in 96-well plates in media. Cell viability was measured using the MTS (Promega, G3580) assay. Cells were treated with siRNA/shRNA/HIF-1α inhibitor under normoxia or hypoxia. Absorption at 450 nm was measured 1 h after addition of MTS reagent to cells, followed by the measurement of absorbance at 450 nm at 0, 24, 48, and 72 h. MDA-MB-468 cells were inoculated into 96-well plates (3.0 × 10^4^ cells per well) and C1QBP expression was silenced. Cells were treated with PTX (0–100 nmol/L) for 24 h followed by the measurement of absorbance at 450 nm to determine the IC50 values of PTX (Solarbio, Cat No. IP0020) under normoxic (20% O_2_) or hypoxic conditions (1% O_2_). Following this, MTS assay data was analyzed to obtain the cell growth inhibition rates exposed to different PTX concentrations. Different PTX concentrations were transformed into base-10 logarithmic scale. Graphpad software was used to analyze the data by XY analyses (nonlinear regression-curve fit and then log (inhibitor) vs. normalized response-variable slope) and estimate IC50 values. We used the following formula to estimate resistance index: Resistance index (RI) of control group = IC50 (control)/IC50 (control). RI of shC1QBP group = IC50 (shC1QBP)/IC50 (control) (Yan et al., 2018).

### Xenograft Studies

MDA-MB-468-control and MDA-MB-468-sh3 cells were transfected with luciferase-containing lentivirus vectors (LV004, constructed by Hanbio company, Shanghai, China). *In vivo* experiments were performed with 4–6 week old female NPG mice (Vitalstar Biotechnology, Beijing, China). Mice were injected 1 × 10^7^ cells subcutaneously and 5 × 10^6^ cells intravenously into the tail vein. 37 NPG mice were used for the *in vivo* studies. For the subcutaneous group, there were 11 mice in control group, including 6 in saline group and 5 in PTX group; 10 mice in shC1QBP group, including 6 in saline and 4 in PTX group, respectively. For the tail vein group, there were 8 mice in control group, including 4 in saline group and 4 in PTX group; 8 mice in shC1QBP group, including 4 in saline group and 4 in PTX group, respectively. For the subcutaneous injection group, we divided the mice randomly into two groups when the average tumor volume reached 150 mm^3^. One group of mice was administered PTX intraperitoneally (Voloshin et al., 2011) (H20020543, 0.5 ml; 20 mg) at a dose of 15 mg/kg, and the control group was administered an equal volume of saline. Tumor volumes after each administration (measured on day 0, day 4, and day 8) were measured. The inhibition rate of tumor growth was calculated as (%) = (Mean tumor volume of saline group - Mean tumor volume of PTX group)/Mean tumor volume of saline group × 100% (Yun et al., 2019; Zhou et al., 2020). Mice were euthanized, tumors removed and photographed. Tumor tissues were embedded in paraffin for pathological examination and hematoxylin-eosin (HE) staining. For the intravenous injection group, we performed luciferase imaging 30 days after injection. Mice were randomly divided into two groups and treated similarly to those in the subcutaneous injection group. All mice were euthanized after the end of treatment and their lungs removed for counting the colonized nodules.

### Transcriptome Sequencing

We sequenced the transcriptome from the treated Hs578T and MDA-MB-468 cells (Hs578T cells: siNC, si1, si2, si3; MDA-MB-468 cells: normoxia, hypoxia) using the Illumina HiSeq Platform. We isolated mRNAs from the total RNA using the oligo (dT) method. Subsequently, mRNAs were fragmented under optimal conditions. First strand and second strand cDNAs were synthesized. We purified and resolved the cDNA fragments using EB buffer for end repair and single nucleotide (adenine) insertion. We then attached the cDNA fragments to adaptors and appropriately sized cDNA fragments were used for PCR amplification. The Agilent 2100 Bioanalyzer and ABI StepOne Plus real-time PCR system were used for quantification and understanding the quality of the libraries. First, low quality reads were filtered out to obtain clean reads and we discovered that more than 20% of base qualities were lower than 10. They included reads with adaptors and unknown bases (more than 5% were “N” bases). The clean reads were then mapped onto a reference genome. This was followed by the prediction of novel genes, and the detection of single nucleotide polymorphisms, insertions and deletions, and spliced gene variants. Finally, we identified the differentially expressed genes among samples and performed clustering analysis with functional annotations.

### Cell–Cell Adhesion Assay

EA.hy926 cells (2 × 10^4^ cells) were stimulated using tumor necrosis factor α (TNFα) (Sino Biology, 10602-HNAE, 1,000 U/ml) for 24 h in 96-well plate. Hs578T (siNC and si3-treated) and MDA-MB-468 (siNC and si3-treated) cells (5 × 10^4^ cells/200 μl) were added to the EA.hy926 cells in each well the following day, and incubated at 37°C for 45 min. Cells were washed twice with FBS-supplemented DMEM. We then added 100 μl of 0.25% rose bengal (RB, Sigma, 3,30,000) per well and incubated at room temperature for 5 min. Cells were washed twice with FBS-supplemented DMEM, followed by the addition of 200 μl of a PBS-ethanol solvent (PBS: 95% ethanol solution at a ratio of 1:1) per well. The cells were then incubated at room temperature for 30 min and the absorbance was measured at 570 nm (Gamble and Vadas, 1988).

HUVECs were seeded in each well of the 96-well plate (2 × 10^4^ cells per well). When the cells reached about 95% confluence, they were treated with TNFα (1,000 U/ml) for 24 h. MDA-MB-468-control and MDA-MB-468-shC1QBP cells were infected with the lentiviral vectors containing GFP (LV001, Constructed by Hanbio Company, Shanghai, China). Next, MDA-MB-468-control or MDA-MB-468-sh C1QBP cells (5 × 10^4^ cells/200 μl) were added onto HUVECs in the 96-well plate and incubated for 30 min at 37°C. After incubation, each well was gently washed three times with PBS to remove non-adherent TNBC cells. The adherent TNBC cells were visualized and photomicrographs were taken using inverted fluorescence microscope with a digital camera. The number of adherent TNBC cells with green fluorescence per field was calculated (Chen et al., 2015). Then 100 μl of 0.25% RB was added per well and incubated at room temperature for 5 min. Cells were washed twice with PBS. The adherent TNBC cells were visualized and photomicrographs. The number of RB-stained adherent cells was calculated.

### Chromatin Immunoprecipitation Assay

Chromatin immunoprecipitation (ChIP) was performed using the Simple ChIP Assay Kit (MILLIPORE, No. 17-371) as per the kit instructions. The DNA-protein complexes were immunoprecipitated using ChIP-grade antibodies against HIF-1α (Novus, NB100-105), P65 (CST, #8242). The precipitated DNA was purified and quantified using real-time PCR and normalized to the inputs. The sequences for binding sites of C1QBP were as follows:

**Table:** 

**Sequences for binding sites**		
C1QBP	Primer 1	F: 5′-TTGGTGACGCCCCTGCTT-3′
		R:5′-GCCGTCCACTCACTGTTTCG-3
	Primer 2	F:5′-CGTATTCTGGGAAGTGTTGTT-3′
		R: 5′-TCACGCATTTCGCTTGGA-3′
	Primer 3	F: 5′-AGCAGGGCTAAGGCGAAGG-3′
		R: 5′-TTCCGGCAGGTGGGTATG-3′
VCAM-1	Primer	F: 5′-CCTTGATGCCCATTTATC-3′
		R: 5′-CTCTAGTCGGTCTGTCTC-3′

### Statistical Analysis

We used GraphPad Prism 6.0 for all statistical analyses. The experimental data were presented as mean ± SD of three independent trials. Two group comparison was analyzed by two-side Student’s *t*-test, and multiple group comparison was analyzed by one-way ANOVA + two-side Dunnett test (when each group compared with a control group) or one-way ANOVA + two-side Tukey test (when each group compared with every other group). Results were considered statistically significant for *p*-values < 0.05.

## Results

### Hypoxia-Induced HIF-1α Upregulates C1QBP in TNBC

**FIGURE 1:**
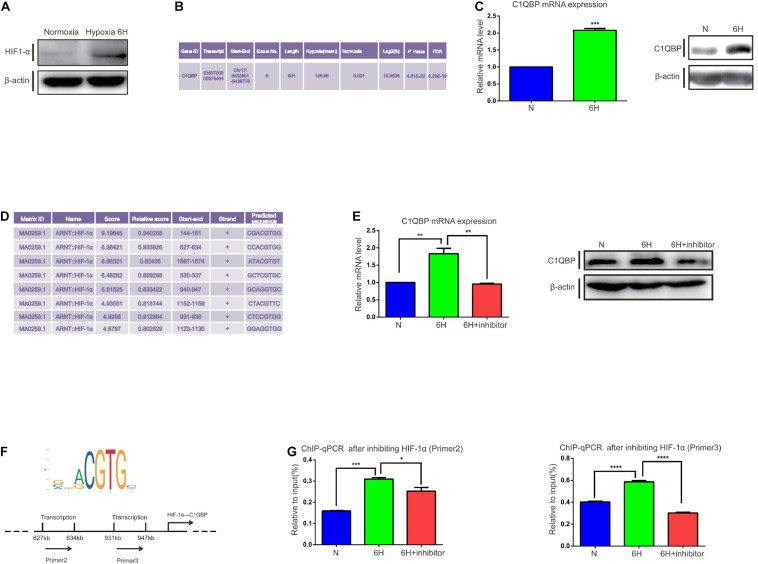
Hypoxia-induced HIF-1α promotes C1QBP upregulation in TNBC. **(A)** MDA-MB-468 cells were exposed to 20% or 1% O_2_ for 6 h. Western blot shows the HIF-1α expression after 6 h of hypoxia; β-actin levels were used as the internal control. **(B)** Transcriptome analysis shows that C1QBP was upregulated during hypoxia. **(C)** MDA-MB-468 cells were exposed to 20% or 1% O_2_ for 6 h. Real-time qPCR and western blot were performed to determine the mRNA and protein levels of C1QBP; β-actin levels were used as the internal control. **(D)** Using the UCSC and JASPAR databases, 200–2,000 bp were selected downstream of the start site in the C1QBP promoter to show the potential binding sites for HIF-1α on C1QBP. **(E)** MDA-MB-468 cells were exposed to 20% O_2_, 1% O_2,_ and 1% O_2_ with the HIF-1α inhibitor (10 μmol/ml) for 6 h, after which mRNA and protein levels were determined using real-time qPCR and Western blot; β-actin levels were used as the internal control. **(F)** The major binding site of HIF-1α on C1QBP (from the JASPAR database): two potential binding sites on the promoter of C1QBP. **(G)**. MDA-MB-468 cells were exposed to 20% O_2_, 1% O_2_, and 1% O_2_ with the HIF-1α inhibitor (10 μmol/ml) for 6 h. ChIP was performed and the presence of the two binding sites was quantitatively determined using real-time qPCR. Three independent experiments were used to represent the mean ± standard deviation values. *P < 0.05, **P < 0.01, ***P < 0.0001, and ****P < 0.0001.

MDA-MB-468 TNBC cells were exposed to 1% O_2_ hypoxic conditions at 3, 6, 9, 12, 24, and 48 h. HIF-1α protein level increased at 6 h under hypoxia (Figure 1A and Supplementary Figure 1A). Subsequently, transcriptome sequencing was performed to investigate whether there was any change in the target transcripts following 6 h hypoxia. Compared to normoxic conditions, 314 genes were upregulated and 257 were downregulated (Supplementary Figure 1D). Gene ontology (GO) (Supplementary Figure 1B) and Kyoto Encyclopedia of Genes and Genomes (KEGG) pathway (Supplementary Figure 1C) analyses were performed. We observed an enrichment of the following terms that were associated with hypoxia: “Cellular response to hypoxia” and “HIF-1 signaling pathway,” providing strong evidence for the successful induction of hypoxia in TNBC cells. Analysis of the differential expression of transcripts (see more details in NCBI database, Project number: PRJNA644755) revealed that the transcript for C1QBP, ENST00000574444, was significantly upregulated in the hypoxia-induced samples (Figure 1B). We have shown that C1QBP is an oncogene which is highly expressed in breast cancer tissues. We also reported C1QBP expression in TNBC and other breast cancers, tested C1QBP correlation with clinicopathological factors and its role in promoting metastasis (Chen et al., 2009; Niu et al., 2015; Wang et al., 2015). However, the association of C1QBP expression with hypoxic conditions and the gene role in TNBC remain also unexplored. Therefore, we addressed C1QBP signaling in the current study.

We then detected changes in the mRNA and protein levels of C1QBP in MDA-MB-468 cells under 1% O_2_ hypoxic conditions at 3, 6, 9, 12, 24, and 48 h. The primers for C1QBP were designed according to the ENST00000574444 transcript. An increase in C1QBP mRNA expression was observed after 6 h of exposure to hypoxia. Protein levels markedly increased at 6 h (Figure 1C and Supplementary Figure 1E), indicating that the mRNA and protein levels of C1QBP increased under hypoxic conditions.

Since C1QBP expression increased under hypoxia, we speculated whether this upregulation was mediated by the HIFs. We used the UCSC^1^ and JASPAR databases^2^ to study the promoter sequences of C1QBP and determine whether there were transcriptional factors that contained binding sites for the C1QBP promoter. We found that HIF-1α possessed several binding sites for the promoter of C1QBP (Figure 1D). HIF-1α is a hypoxia-inducible transcription factor and its aberrant overexpression promotes the development of breast cancer by, for example, activating multiple steps in the metastatic cascade (Gilkes et al., 2014; Liu et al., 2015). These data suggested that C1QBP might be affected by hypoxia-induced HIF-1α expression.

Then, shRNA targeting HIF-1α or HIF-1α inhibitor (2-methoxyestradiol) were used. A lentiviral vector was successfully constructed and transfected into MDA-MB-468 cells to silence HIF-1α expression. Western blot and qRT-PCR were used to determine the knockdown efficiency (Supplementary Figure 2C). HIF-1α inhibitor was also used to block the function of HIF-1α and decreased HIF-1α mRNA levels (Supplementary Figure 2D). We first performed cell viability assay to compare the inhibitory effects between shRNA and HIF-1α inhibitor on Hs578T and MDA-MB-468 cells under normoxia or hypoxia. The results indicated that application of HIF-1α inhibitor exhibited stronger inhibitory activities than that of shHIF-1α inhibitor (Supplementary Figure 2A). MDA-MB-468 cells were then exposed to hypoxic environments and treated with the HIF-1α inhibitor for 6 h to block its function. Both the mRNA and protein levels of C1QBP increased under hypoxic conditions and decreased upon treatment with the HIF-1α inhibitor as expected (Figure 1E). Accordingly, we further detected the protein level of vascular endothelial growth factor (VEGF), von Hippel-Lindau tumor suppressor (VHL), E-cadherin 1 (E-cad), and β-tubulin, that are reported to be regulated by hypoxia and/or HIF-1α (Bordji et al., 2014; Ning et al., 2018; Ibuki et al., 2020; Urrutia et al., 2020; Yang et al., 2020) (Supplementary Figure 2B). Based on the data from the JASPAR database, we generated several primers for HIF-1α containing multiple binding sites on the C1QBP promoter (Figure 1F). ChIP-qPCR assay was performed and the results indicated that the inhibition of HIF-1α decreased the binding of HIF-1α to the promoter of C1QBP (Figure 1G).

Since HIF-1α influences C1QBP expression under hypoxic conditions, we analyzed whether this phenomenon existed in normoxic conditions. The mRNA levels of C1QBP decreased after using shHIF-1α or HIF-1α inhibitor (Supplementary Figures 2E,F). However, unlike the decreased tendency under hypoxia, the protein levels did not show obvious changes under normoxia (Supplementary Figure 2G). Subsequently, ChIP-qPCR was performed and the results indicated that inhibiting HIF-1α decreased the binding of HIF-1α to the promoter of C1QBP (Supplementary Figures 2H,I). Using the results from The Cancer Genome Atlas database (TCGA) database, we found C1QBP mRNA levels to increase in breast cancer tissues concomitantly with the increasing levels of HIF-1α (Supplementary Figure 2J).

Together, all these data suggested that hypoxia induced the expression of C1QBP mediated by the HIF-1α transcription factor in TNBC cells.

### Knockdown of C1QBP Inhibits TNBC Migration, Invasion, and Chemoresistance to PTX *in vitro*

**FIGURE 2:**
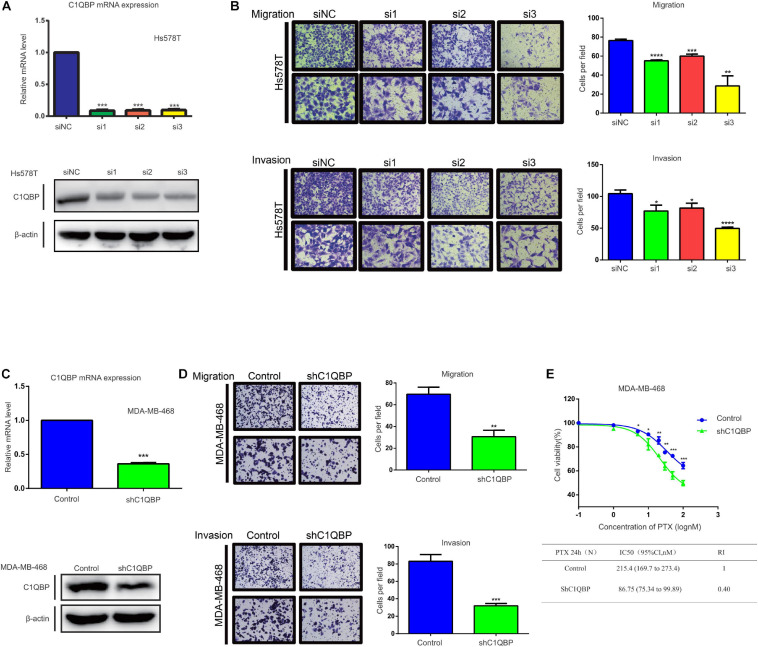
Knockdown of C1QBP inhibits TNBC migration, invasion, and chemoresistance to paclitaxel *in vitro*. **(A)** Knockdown of C1QBP was confirmed using real-time qPCR and western blot after Hs578T cells were transfected with siRNA. **(B)** Migration and invasion analysis indicates differential cell motilities in Hs578T cells. **(C)** The knockdown efficiency of C1QBP was confirmed using real-time qPCR and western blot in MDA-MB-468-shC1QBP cells. **(D)** Migration and invasion analysis indicates differential cell motilities in MDA-MB-468 cells. **(E)** Upper: cell viabilities were determined in MDA-MB-468-control and shC1QBP cells incubated with PTX for 24 h by MTS. Lower: comparison of the IC50 values and resistance indices of control and shC1QBP groups. Three independent experiments were used to represent the mean ± standard deviation values. *P < 0.05, **P < 0.01, ***P < 0.0001, and ****P < 0.0001.

In the present study, we used two TNBC cells, Hs578T and MDA-MB-468, to understand the correlation between C1QBP expression and migration, invasion. Firstly, three siRNAs were designed for transient transfection into Hs578T cells to reduce C1QBP gene expression. Figure 2A shows the mRNA and protein levels to determine the efficiency of knockdown of C1QBP after transient transfection. We next performed cell viability experiments to evaluate the effect of C1QBP on Hs578T cells. The result showed that unlike under normoxia, silencing C1QBP inhibited Hs578T cell proliferation under hypoxia (Supplementary Figure 3D). Transwell experiments indicated that knocking down C1QBP significantly reduced the capacity for migration and invasion of Hs578T cells; this phenotype became more pronounced after transfecting with the si3 (Figure 2B). Given that si3 was most effective at inhibiting migration and invasion, we used it to block C1QBP expression in MDA-MB-468 cells (Supplementary Figure 3A), which resulted in similar phenotypes (Supplementary Figures 3B,C). Based on the results using si3, we generated a stable C1QBP knockdown cell line with shC1QBP (Figure 2C) and the migration, invasion capacity was observed to be impaired (Figure 2D). Then cell viability experiments were performed to evaluate the effect of shC1QBP on MDA-MB-468 cells. Similar result was observed in MDA-MB-468 cells (Supplementary Figure 3E). To determine whether silencing C1QBP changed the chemoresistance of cells to PTX, we measured the viability of shC1QBP cells upon treatment with different concentrations of PTX. Compared to the control, cell viability was inhibited in the shC1QBP cells and the IC50, resistance index was lower (Figure 2E). These results indicated that blocking C1QBP inhibited the migration, invasion and chemoresistance of TNBC cells to PTX *in vitro.*

### Knockdown of C1QBP Hinders *in vivo* TNBC Lung Colonization and Chemoresistance to PTX

**FIGURE 3:**
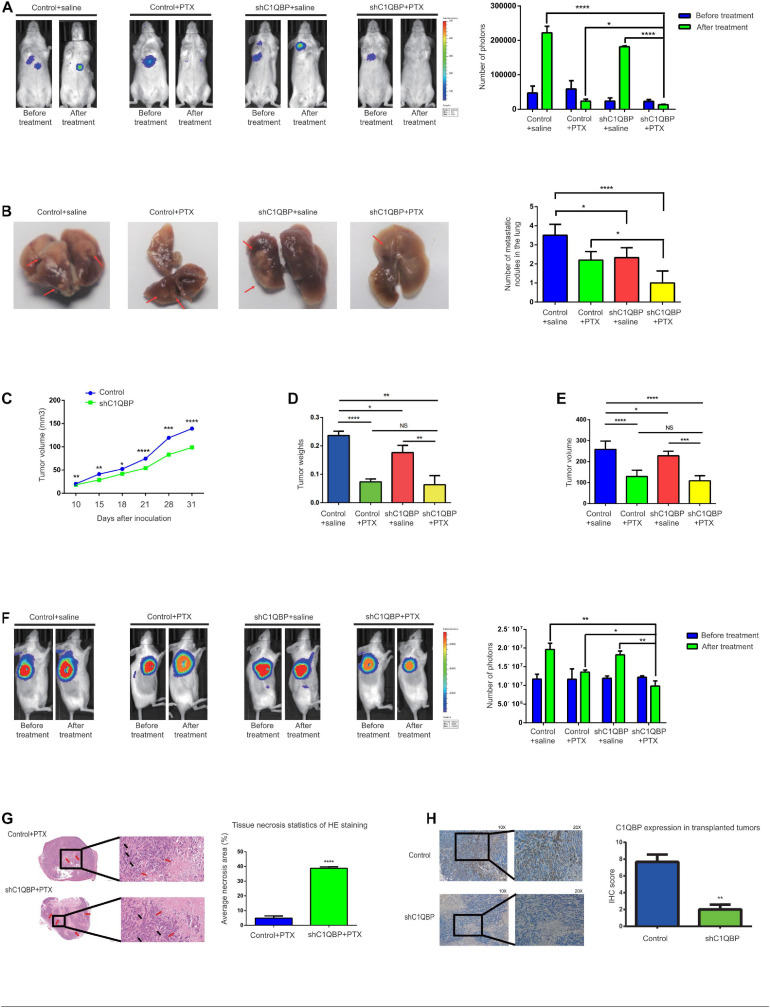
Knockdown of C1QBP hinders *in vivo* TNBC lung colonization and chemoresistance to paclitaxel. **(A)** Representative IVIS imaging of lungs in control and shC1QBP mice were exhibited before and after treatment. Luci-labeled tumor cells were injected into tail veins of mice. Consequently, tumor cells were located in the area where the lung tissue was illuminated. Tumor photon numbers were also assessed. **(B)** Representative colonization of the lungs was quantified, respectively. The white translucent area was recognized as lung colonization by tumors. Lung colonization by tumors is indicated by a red arrow. **(C)** Stable control and C1QBP-depleted cells (1 × 10^7^) were injected subcutaneously into NPG mice. Tumor volumes were measured. **(D)** The control and shC1QBP groups were treated with PTX at a dose of 15 mg/kg or saline three times every 4 days after the average tumor volume reached 150 mm^3^. The weights of tumors were measured. **(E)** The volumes of tumors were measured. **(F)** Representative IVIS imaging of tumor cells in subcutaneous control and shC1QBP mice were exhibited before and after treatment. The numbers of photons of tumors were measured. **(G)** HISTECH Pannoramic250 analysis of HE staining and necrosis in the subcutaneous neoplastic tissues of PTX-treated control or shC1QBP mice. Left panel shows HE-stained tissues treated with PTX and control (upper) or shC1QBP (lower). Black arrows show tumor cells and red arrows show necrotic regions. Right panel shows the quantification for the same. **(H)** IHC shows the protein levels of C1QBP in the subcutaneous neoplastic tissues after treatment with control or shC1QBP. The left panel represents the expression of C1QBP with control (upper; high expression) and shC1QBP (lower; low expression). The right panel shows the quantification of IHC scores. Three independent experiments were used to represent the mean ± standard deviation values. *P < 0.05, **P < 0.01, ***P < 0.0001, and ****P < 0.0001.

We further validated the *in vivo* effects of C1QBP in TNBC. Both the MDA-MB-468-control and MDA-MB-468-shC1QBP cell lines were transfected with lentivirus luciferases (Supplementary Figure 4A) and injected into NPG mice via the tail vein and subcutaneous routes, respectively. For the tail vein-injected group, fluorescence imaging showed that C1QBP-depleted cells significantly reduced lung colonization in mice (Figure 3A and Supplementary Figure 4B). Then we treated the control and shC1QBP mice with saline or PTX, respectively. PTX treatment significantly inhibited the colonization of tumor cells in the lung (Figure 3A and Supplementary Figure 4B). Moreover, the numbers of tumors were the lowest in shC1QBP+PTX group compared to all other groups (Figure 3B), indicating that PTX combined with silencing of C1QBP expression reduced the lung colonization by tumors. Then we performed the HE staining of the lung tissues. For saline-treated groups, the size and number of the colonization in the lung are larger in control group than that in shC1QBP group. For PTX treated mice, the size and number of the colonization in the lung are larger in control group than that in shC1QBP group (Supplementary Figure 5A). Immunohistochemistry (IHC) staining also showed that the expression of ki67 was higher in control+saline group than those in shC1QBP+saline group. In shC1QBP+PTX group, ki67 showed the lowest expression (Supplementary Figure 5B), indicating that silencing C1QBP combined with PTX might play a better role in inhibiting proliferation. These results were in line with the fluorescence imaging studies (Figure 3A).

As application of subcutaneous model has advantage in monitoring the growth of tumor, tumor growth curve detection and evaluation of drug efficacy (Masuelli et al., 2017; Ireson et al., 2019; Lee et al., 2020), we chose subcutaneous model to evaluate the inhibition effect by knocking down C1QBP or shC1QBP combined with PTX treatment. For the subcutaneously injected group, the volumes of tumors formed by the control cells were significantly greater than those from cells expressing lower levels of C1QBP (Figure 3C), suggesting that silencing C1QBP restrained cell proliferation in mice. After the xenograft-TNBC models were established and the average tumor volumes reached 150 mm^3^ (in both control and shC1QBP groups), PTX resistance was subsequently tested in association with C1QBP depletion. Control and shC1QBP-transfected mice were treated with PTX using intraperitoneal injection. For the shC1QBP+PTX group, the average inhibitory rates were much higher compared to those of the control+PTX group (day 4 after treatment: control vs. shC1QBP: 0.58% vs. 22.60%; day 8 after treatment: control vs. shC1QBP: 27.20% vs. 39.76%; day 12 after treatment: control vs. shC1QBP: 50.08% vs. 52.24%). Tumor weights and volumes were measured after the last PTX treatment. The results showed that the tumor weights/volumes of the shC1QBP+saline group were lower than that of control+saline group. No significant difference was observed between data in shC1QBP+PTX and control+PTX group (Figures 3D,E and Supplementary Figure 4C). However, comparing the number of photos in each group, it was found that shC1QBP+PTX mice contained the lowest number of tumor nodes than mice in other groups (Figure 3F and Supplementary Figure 4C). Moreover, HE staining of the subcutaneous tumorigenic tissues showed that the areas of necrosis in the shC1QBP+PTX mice were significantly greater than those in the control+PTX mice (Figure 3G), suggesting that knocking down C1QBP impaired the resistance of TNBC cells to PTX *in vivo*. Besides, IHC staining confirmed the differential expression of C1QBP in the control and shC1QBP mice in *in vivo* experiments (Figure 3H). The ki67 staining showed the lowest expression in shC1QBP+ PTX group mice (Supplementary Figure 5C). These results strongly indicated that depletion of C1QBP inhibited the lung colonization, proliferation and chemoresistance of TNBC cells *in vivo*.

### Depletion of C1QBP Downregulates VCAM-1 Expression in TNBC via P65 in the Nucleus

**FIGURE 4:**
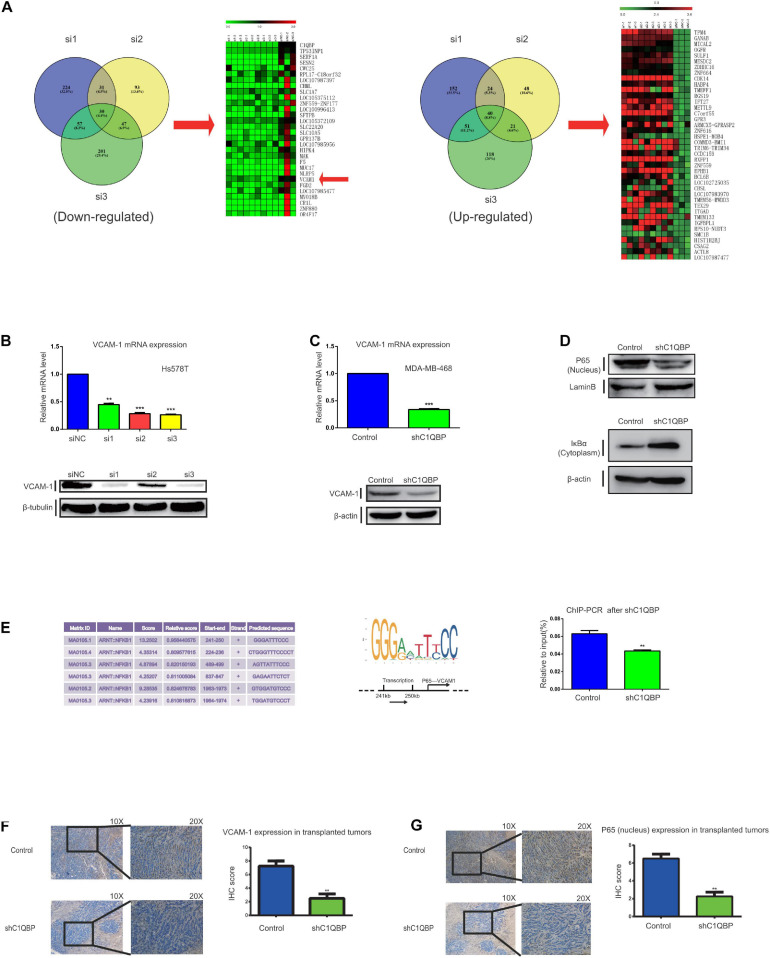
Depletion of C1QBP downregulates VCAM-1 expression in TNBC via P65 in the nucleus. **(A)** Heatmap of the 30 commonly downregulated and 40 upregulated genes in the si1, si2, and si3 groups as compared to siNC were selected. Down- and upregulated genes from the si1, si2, and si3 groups were selected and Venn diagrams were generated to find the commonly regulated genes. **(B)** Real-time qPCR and western blot were used to determine the mRNA and protein levels of VCAM-1 after transfection with siC1QBP in Hs578T cells for 48 h. **(C)** Real-time qPCR and western blot were used to determine the mRNA and protein levels of VCAM-1 after transfection with shC1QBP in the MDA-MB-468 cells. **(D)** Nuclear and cytoplasmic proteins were extracted from MDA-MB-468 control and shC1QBP cells. Western blot was performed to determine the protein levels of P65 and IκBα; LaminB or β-actin was used as internal controls, respectively. **(E)** Potential binding sites of P65 to the promoter of VCAM-1 and the major binding site of P65 to the promoter of VCAM-1 as obtained from the JASPAR database are shown. MDA-MB-468 control and shC1QBP cells were used to perform ChIP-qPCR. **(F)** IHC staining shows the protein levels of VCAM-1 in subcutaneous neoplastic tissues upon treatment of control or shC1QBP groups. The left panel represents the expression of VCAM-1 in tumors of control (upper panel; high expression) or shC1QBP mice (lower panel; low expression). The right panel shows the quantification of IHC scores. **(G)** IHC staining shows the P65 protein levels in the subcutaneous neoplastic tissues. The upper panel represents the expression of P65 upon treatment with control (high expression) and the lower panel represents the expression in shC1QBP tissues (low expression). The right panel shows the quantification of IHC scores. Three independent experiments were used to represent the mean ± standard deviation values. **P < 0.01 and ***P < 0.0001.

Since C1QBP expression was associated with metastasis and chemoresistance, we subsequently explored the effectors downstream of C1QBP in TNBC. Transcriptome sequencing was performed after depleting C1QBP using siRNA in Hs578T cells. Volcano plots (Supplementary Figure 6A) were used to determine the variation in gene expression between the siNC and siC1QBP groups (see more details in NCBI database, Project number: PRJNA644772). Functional GO (Supplementary Figure 6B) and KEGG pathway analysis (Supplementary Figure 6C) of the differentially expressed genes were performed. Pathway involving “Basal cell carcinoma” was markedly enriched in the si3 group. Compared to the siNC group, the numbers of upregulated genes in the si1, si2, and si3 groups were 346, 219, and 314, respectively. The numbers of downregulated genes in the si1, si2, and s3 groups were 465, 312, and 454, respectively. We then analyzed the intersection between the up- and downregulated genes in the si1, si2, and si3 groups; there were 30 genes that were commonly downregulated and 40 upregulated in the three siRNA groups compared to those in the siNC group (Figure 4A and Supplementary Figures 6D,E).

Considering the established metastasis-promoting role of VCAM1, we selected this gene for further analysis. VCAM-1 is a member of the immunoglobulin superfamily and a cytokine-induced endothelial adhesion protein (Labrousse-Arias et al., 2017). VCAM-1 promotes the progression of tumors and plays an important role in the metastasis of breast cancer to the lungs, thereby making it a potential therapeutic target for inhibiting metastasis (Chen and Massague, 2012; Shokeen et al., 2012; Garmy-Susini et al., 2013; Dan et al., 2016). To verify the correlation between the expression of C1QBP and VCAM-1, we detected mRNA and protein levels after treatment with siC1QBP in Hs578T cells (Figure 4B); the expression of VCAM-1 was significantly downregulated and similar results were seen in MDA-MB-468 cells after using si3 (Supplementary Figure 7A) or shC1QBP (Figure 4C) to silence the expression of C1QBP. During metastasis to the lungs in breast cancer, VCAM-1 recognizes the α-subunit, interacts with 4-integrin on monocytes, and enhances the formation of metastatic tumor cells in lung tissues (Dan et al., 2016). VCAM-1 is involved in the recruitment of inflammatory cells to the injured tissue; this process also depends on the interaction between VCAM-1/α4β1 and VCAM-1/α4β7 (Shokeen et al., 2012; Garmy-Susini et al., 2013). Subsequently, we used TNFα to stimulate EA.hy926 endothelial cells (Brezinschek et al., 1996); α4, β1 and β7 were successfully induced by the upregulation of mRNA levels (Supplementary Figure 7B). Both the MDA-MB-468-control and MDA-MB-468-shC1QBP cell lines were transfected with lentivirus GFP (Supplementary Figure 7D). Cell-cell adhesion assays showed that the depletion of C1QBP inhibited the capacity of Hs578T and MDA-MB-468 cells to adhere to EA.hy926 cells or HUVECs that were induced for 24 h by TNFα (Supplementary Figures 7C,E). Moreover, IHC staining of the subcutaneous tumorigenic tissues revealed that the protein levels of VCAM-1 in the shC1QBP group were lower than those in the control group (Figure 4F). Using the results from the TCGA database, we found C1QBP mRNA levels in breast cancer tissues were associated with the levels of VCAM-1 (Supplementary Figure 7F). All these findings strongly indicated that the loss of C1QBP blocked the expression of VCAM-1 in TNBC.

We then investigated how C1QBP influenced the expression of VCAM-1 in TNBC. According to the pathway enrichment analysis using the data from transcriptome sequencing, VCAM-1 (downregulated) is a downstream molecule in the NF-κB signaling pathway (Supplementary Figure 8A). Previous studies have reported that the NF-κB signaling pathway is a major regulator of VCAM-1 in many cell types (Lin et al., 2015). Therefore, we hypothesized that C1QBP regulates VCAM-1 expression by affecting the NF-κB signaling pathway. The canonical NF-κB pathway is activated by heterodimers of p50 and P65 (Oeckinghaus and Ghosh, 2009). Nuclear P65 is a transcription factor and an important component of the NF-κB pathway that can be targeted for drug discovery and development (Giridharan and Srinivasan, 2018). It has also been reported that nuclear translocation of P65 is sufficient for VCAM-1 expression (Zerfaoui et al., 2008; Cartee et al., 2012). Western blot analysis showed that silencing C1QBP decreased the P65 protein levels in the nucleus of MDA-MB-468 cells (Figure 4D). The activation of NF-κB also depends on phosphorylation-induced ubiquitination of the IκB proteins (Giridharan and Srinivasan, 2018). Further experiments revealed that the levels of nuclear factor-kappa B inhibitor alpha (IκBα) in the cytoplasm increased upon knocking down C1QBP in the cells (Figure 4D). TNF, which acts as a target gene of NF-κB signal, also decreased after in shC1QBP cells (Supplementary Figure 8B), indicating that C1QBP may regulate the activity of the NF-kB signal pathway. Since P65 is a transcription factor in the nucleus, we used the USCS and JASPAR databases to determine the binding sites of P65 in the promoter of VCAM-1 (Figure 4E). Based on these data, we designed the necessary primers and performed ChIP-qPCR; the results of this assay showed that knocking down C1QBP decreased the binding of P65 to the promoter of VCAM-1 (Figure 4E).

To further confirm the mechanism of action of P65 *in vivo*, we collected tumor tissues after subcutaneously injecting control or sh3 cells. The protein levels of P65 decreased as expected (Figure 4G). IHC staining showed that the expression of VCAM-1 were higher in control+saline group than those in shC1QBP+saline group. Compared to control+saline group, the expression of VCAM-1 did not show obvious decline in control+ PTX mice. However, in shC1QBP+PTX group, VCAM-1 showed weak expression (Supplementary Figure 8C), indicating that silencing C1QBP combined with PTX might play a better role in inhibiting adhesion abilities of TNBC. Still, we observed similar results of VCAM-1 IHC staining to the intravenous mice (Supplementary Figure 8D).

Thus, C1QBP depletion impaired NF-κB signaling and downregulated VCAM-1 expression by lowering the protein levels of P65 in the nucleus of TNBC cells.

### Depletion of C1QBP Inhibits Hypoxia-Induced Activation of PKC-NF-κB-VCAM-1 Signaling

**FIGURE 5:**
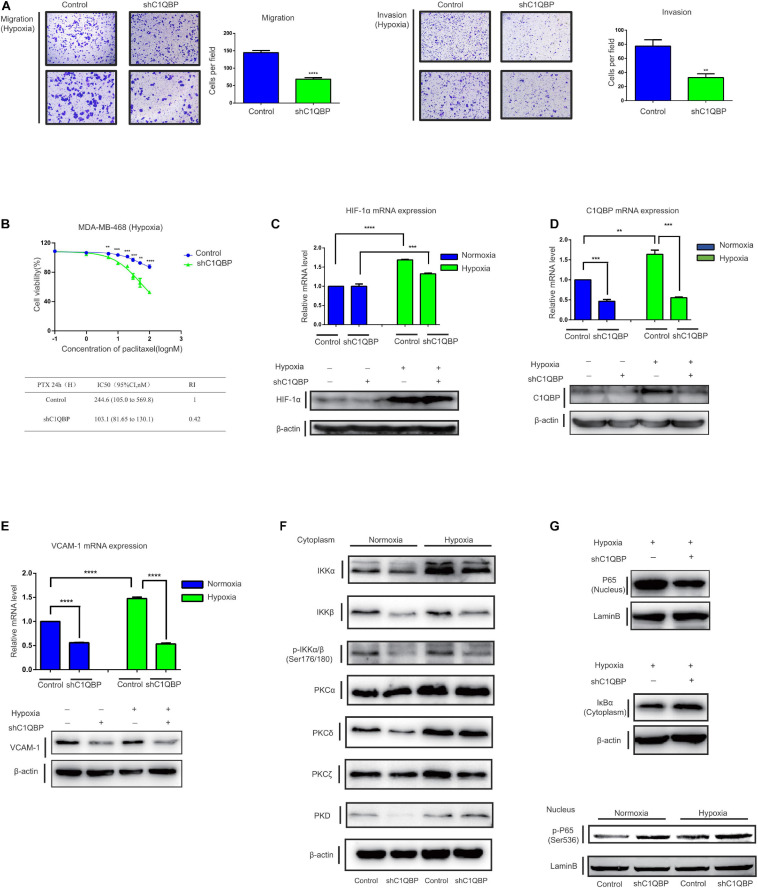
Depletion of C1QBP inhibits hypoxia-induced activation of the PKC–NF-κB–VCAM-1 signaling pathway. **(A)** MDA-MB-468 control and shC1QBP stable cells were exposed to 1% O_2_ for 24 h and the potential for migration (left) and invasion (right) were measured using transwell assay. **(B)** MDA-MB-468-control and shC1QBP cells incubated with PTX under exposure to 1% O_2_ for 24 h. The upper panel shows cell viabilities using MTS assay. The lower panel shows the comparison of the IC50 value and resistance index of control and shC1QBP samples. **(C)** MDA-MB-468 control and shC1QBP stable cell lines were exposed to 20% or 1% O_2_ for 6 h; subsequently, the mRNA and protein expression of HIF-1α were determined. **(D)** MDA-MB-468 control and shC1QBP stable cell lines were exposed to 20% or 1% O_2_ for 6 h. Subsequently, the mRNA and protein expression of C1QBP were determined. **(E)** MDA-MB-468 control and shC1QBP stable cell lines were exposed to 20% or 1% O_2_ for 6 h. Subsequently, the mRNA and protein expression of VCAM-1 were determined. **(F)** MDA-MB-468 control and shC1QBP stable cells were exposed to 20% or 1% O_2_ for 6 h followed by the isolation of cytoplasmic proteins. The expression of proteins involved in PKC and NF-κB signaling was detected. **(G)** MDA-MB-468 control and shC1QBP stable cell lines were exposed to 20% or 1% O_2_ for 6 h followed by the isolation of nuclear and cytoplasmic protein. The protein levels of P65, IκBα, and p-P65 (Ser536) were detected. Three independent experiments were used to represent the mean ± standard deviation values. **P < 0.01 and ***/****P < 0.0001.

Since the knockdown of C1QBP downregulated TNBC metastasis, chemoresistance capabilities, and VCAM-1 expression via P65 in normoxic conditions, we attempted to determine whether this effect still persisted under hypoxia. As C1QBP influenced TNBC cell proliferation under hypoxia, we performed transwell experiments along with mitomycin C. The results showed that the loss of C1QBP inhibited the migration and invasion properties of MDA-MB-468 and Hs578T cells under hypoxic conditions (Figure 5A and Supplementary Figure 9A). MDA-MB-468-control and MDA-MB-468-shC1QBP cells were treated with different concentrations of PTX. IC50 values, and resistance indices decreased in the C1QBP knockdown cells significantly (Figure 5B), as was expected based on our previous *in vivo* results. Similar results were observed in Hs578T cells (Supplementary Figure 9B).

MDA-MB-468-control and MDA-MB-468-shC1QBP cells were then exposed to normoxic or 1% O_2_-containing hypoxic conditions. Compared to those under normoxia, HIF-1α mRNA and protein levels increased upon exposure to hypoxia (Figure 5C). The levels of C1QBP and VCAM-1 in control cells were upregulated in both normoxic and hypoxic conditions, and were both downregulated in the sh3 cells (Figures 5D,E). These data indicated that depletion of C1QBP reduced the metastatic and chemoresistance abilities and inhibited the expression of VCAM-1 of TNBC cells under hypoxia.

Protein kinase C isoforms play a crucial role in carcinogenesis (Isakov, 2018) and are upstream activators of NF-κB signaling (Li and Luan, 2018; Cao et al., 2019). We then determined the expression of proteins in the protein kinase C-nuclear factor-kappa B (PKC-NF-κB) pathway under normoxic and hypoxic conditions. Our study confirmed that IKKα, PKCα, PKCδ, and PKCζ were upregulated under hypoxia (Figure 5F). It has been shown previously that PKCa (Qureshi-Baig et al., 2019) and PKCδ (Ozpolat et al., 2007) are key markers associated with hypoxia. The activation of NF-κB was linked to hypoxia (Wu et al., 2019). We also found that IKKa, which is a hallmark effector of NF-κB pathway, was up-regulated in hypoxia, indicating that NF-κB signal was activated. The expression of IKKα, IKKβ, p-IKKα/β, PKCδ, and PKCζ was inhibited when C1QBP expression was silenced (Figure 5F), indicating that shC1QBP can inhibit the hypoxia-induced activation of PKC-NF-κB signal. As expected, the protein levels of P65 decreased and the expression of IκBα increased in the cytoplasm (Figure 5G). The mRNA level of TNF decreased (Supplementary Figure 9C). We also found that MDR1, another downstream molecule of NF-κB signal (Fan et al., 2020), was downregulated in shC1QBP cells under hypoxia (Supplementary Figure 9D). As MDR1 causes drug resistance (Wang et al., 2020), we further explored whether C1QBP regulated chemoresistance of cells to PTX via MDR1 expression. MDR1-overexpression plasmid was transfected in MDA-MB-468 control and shC1QBP cells (Supplementary Figure 9E). Our result showed that re-expression of MDR1 overcome increased PTX sensitivity of C1QBP antagonized cells, indicating that C1QBP might regulate TNBC cell chemoresistance via MDR1 protein (Supplementary Figure 9F). Moreover, the levels of p-P65 (Ser536) in the nucleus increased after silencing C1QBP under both normoxic and hypoxic conditions (Figure 5G). These results collectively suggested that depletion of C1QBP decreased hypoxia-mediated TNBC cell metastasis and chemoresistance and inhibited hypoxia-induced activation of PKC–NF-κB–VCAM-1 signaling.

### Complement 1q Binding Protein Level Correlates With VCAM-1 and P65 Expression in TNBC Patient Tissues

**FIGURE 6:**
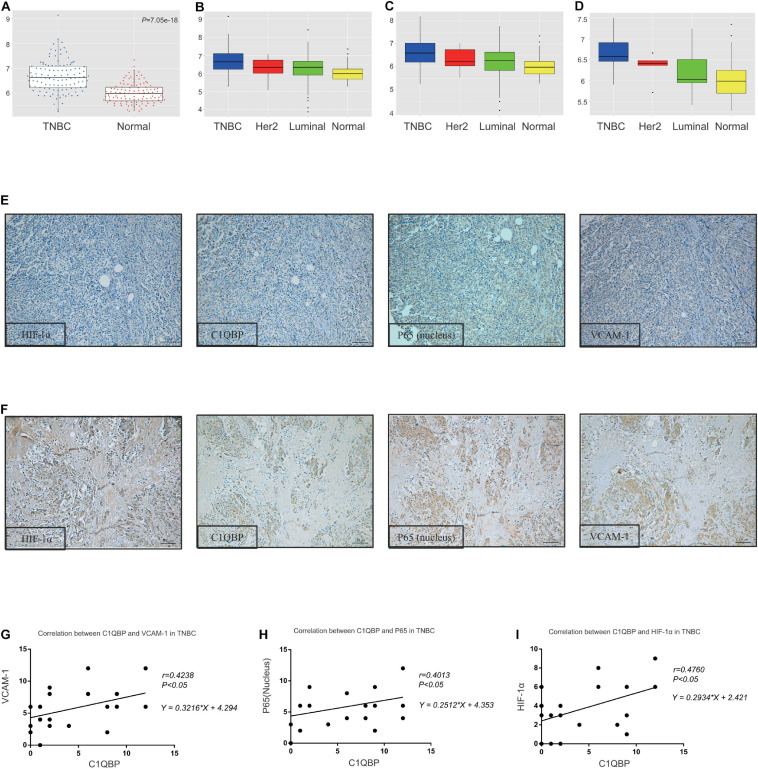
The C1QBP level correlates with VCAM-1 and P65 expression in TNBC patient tissues. **(A)** Comparison of the expression of C1QBP in TNBC and normal tissues in the TCGA database. **(B)** Comparison of the expression of C1QBP in TNBC and other subtypes of breast cancer patients in the TCGA database. **(C)** Comparison of the expression of C1QBP in TNBC and other subtypes of metastatic breast cancer patients in the TCGA database. **(D)** Comparison of the expression of C1QBP in TNBC and other subtypes of dead breast cancer patients in the TCGA database. **(E)** Representative IHC images of low levels of HIF-1α, C1QBP, VCAM-1, and P65 in patients with TNBC. **(F)** Representative IHC images of high levels of HIF-1α, C1QBP, VCAM-1, and P65 in patients with TNBC. **(G)** Correlation analyses of the IHC staining scores of C1QBP and VCAM-1 levels in the tumor cells of patients with TNBC. **(H)** Correlation analyses of the IHC staining scores of C1QBP and P65 levels in the tumor cells of patients with TNBC. **(I)** Correlation analyses of the IHC staining scores of C1QBP and HIF-1α levels in the tumor cells of patients with TNBC.

Based on these findings, we performed analyses of data from the TCGA database; the mRNA levels of C1QBP increased significantly in TNBC tissues compared to those in normal tissues (Figure 6A). Further analysis using PAM50 typing showed that C1QBP expression in TNBC was the highest among all the molecular subtypes of breast cancer (Figure 6B). Similarly, among breast cancer patients with metastasis or dead, the expression level of C1QBP in TNBC is still higher than that in other subtypes (Figures 6C,D). These findings strongly indicated that C1QBP might be a potential specific biomarker of TNBC in patients.

Since HIF-1α upregulates C1QBP and C1QBP regulates the expression of VCAM-1 and P65 in TNBC cells both *in vitro* and *in vivo*, tissue microarray specimens from 271 breast cancer patients were used to detect the four indicators mentioned above, using IHC staining. The positive and negative reactions for these proteins in TNBC patients were consistently obtained (Figures 6E,F). The correlation between C1QBP levels and those of VCAM-1, P65, and HIF-1α was analyzed based on IHC scores. C1QBP levels were associated with the expression of VCAM-1, P65 and HIF-1α in patients with breast cancer patients (Supplementary Figures 10A–C), especially in TNBC (Figures 6G–I). Further analysis revealed that the expression of HIF-1α, along with VCAM-1 and P65 levels, correlated well with each other in breast cancer (Supplementary Figures 10D–F) and TNBC tissues (Supplementary Figures 10G–I), and was in accordance with previous studies (Zerfaoui et al., 2008; Oeckinghaus and Ghosh, 2009; Cartee et al., 2012; Guan et al., 2014; Liang et al., 2019). These clinical data provided strong evidence confirming that C1QBP expression was correlated with the expression of HIF-1α, VCAM-1, and P65.

## Discussion

Fogal et al., 2010 first find out that C1QBP promotes tumorigenicity of tumor cells. They infer a role for mitochondrial integrity and respiration that may influence cancer cell viability (Fogal et al., 2010). Metastasis remains one of the most critical situations that reduce TNBC patient survival. The metastatic processes of tumor cells include local migration, invasion, and the colonization of secondary sites away from primary tissues. These steps are controlled by several signaling and transcription pathways (Chaffer and Weinberg, 2011); the signaling pathways that are responsible for the regulation of metastasis are yet to be fully understood. Fogal et al. show that C1QBP knockdown alters tumor cell metabolism, impairs cell growth and increases cell death, influences synthesis of mitochondrially encoded proteins and inhibits tumor maintenance and malignancy. C1QBP regulates the balance between OXPHOS and glycolysis and therefore influence tumorigenic and metastasis of MDA-MB-231 cells both in vitro and in vivo, highly indicating that C1QBP may serve as an oncogene and promote metastasis of TNBC (Fogal et al., 2010). Our results were consistent with their findings. Depletion of C1QBP inhibited cell proliferation and metastasis of TNBC cells, suggesting that the decreased tumorigenicity of TNBC cells might be caused by mitochondrial dysfunction and glucose metabolism disorder. Previous studies have demonstrated that hypoxia-mediate OXPHOS and glycolysis of tumor cells (Wu et al., 2020). Whether C1QBP regulates OXPHOS and glycolysis under hypoxia and its potential mechanisms needs further investigation.

Here, we identified C1QBP, a gene that we have previously studied, participated in regulating metastasis of TNBC cells under hypoxia. Using transwell assay, we found that si3 demonstrated the strongest inhibition of migration and invasion of TNBC cells compared with the other two siRNA (Figure 2B and Supplementary Figure 9A). We also found out that C1QBP regulated the levels of the metastasis-associated protein VCAM-1. Considering the inhibition of downstream target gene VCAM-1, si3 also significantly inhibited VCAM-1 mRNA and protein levels (Figure 4B and Supplementary Figure 7A). Conclusively, Si3 demonstrated significant antitumor effect in TNBC cells. Therefore, we designed the si3-corresponding shRNA and mainly used this shRNA alone in subsequent animal and in vitro experiments. C1QBP also modulated TNBC cell-endothelial cell adhesion (Supplementary Figures 7C,E). However, the underlying mechanism of adhesion of TNBC cells to endothelial cells is not well understood and requires further investigation.

Besides, according to the RNA-seq data, we detected the tumor protein p53 inducible nuclear protein 1 (TP53NIP1), a p53 target gene that induces cell growth arrest and apoptosis by modulating p53 transcriptional activity, was significantly downregulated after cells transfected with siC1QBP (Figure 4A), suggesting that C1QBP might be involved in regulation of apoptosis and stress of TNBC cells. Studies have proved that TP53NIP1 acts as a suppressor in several tumors (Seux et al., 2011; Wei et al., 2012; Ng et al., 2017; Chen et al., 2020), while it can also promote cancer progression (Li et al., 2019). Further testing of TP53NIP1 effect in TNBC is required. As C1QBP regulates the balance between OXPHOS and glycolysis in TNBC (Fogal et al., 2010), the relationship between C1QBP and TP53NIP1 in tumor cell stress needs further investigation.

HIF-1α was discovered in mammalian cells in cultures under reduced O_2_ pressure; it is necessary for the activation of transcription in cells responsive to hypoxia (Wang et al., 1995). The transcriptional factor HIF-1α regulates various genes in the hypoxic microenvironment in tumors, and high levels are linked to high-grade malignancies (Keith et al., 2011), indicating that further research on cancers associated with hypoxia is of great importance. Fogal et al., 2008 show that C1QBP serves as a new marker of tumor cells and tumor-associated macrophages/myeloid cells in hypoxic/metabolically deprived areas of tumors (Fogal et al., 2008). Their findings highly indicate that the expression of C1QBP might be regulated by hypoxia. In this report, the TNBC cell line MDA-MB-468 was used to study various transcriptional changes involved in the response to hypoxia (1% O_2_). Our results suggested that TNBC cells were sensitive to reduced O_2_ content (Figure 1A and Supplementary Figure 1A) and that the mRNA and protein levels of C1QBP were correspondingly upregulated (Figure 1C and Supplementary Figure 1E), indicating that genes like might be abnormally expressed or active in tumor-associated microenvironments. These results might explain why C1QBP was overexpressed in TNBC patients. Studies have confirmed that ZNF32 and c-Myc regulate C1QBP expression in liver cancer (McGee and Baines, 2011) and gliomas (Fogal et al., 2015). Further experiments are required to understand the expression of C1QBP at the transcriptional modification level in TNBC cells.

Previous studies have shown that hypoxia is associated with resistance to chemotherapy (Liang et al., 2019). Further investigation revealed that chronic exposure of TNBC cells to hypoxia for 48 h continuously increases the expression of HIF-2α and promotes TNBC cell resistance to PTX (Yan et al., 2018). C1QBP stabilizes the MRE11 protein by forming an MRC complex with MRE11/RAD50 and inhibiting MRE11 exonuclease activity, thereby demonstrating that C1QBP plays a crucial role in the DNA damage response and serves as a potential target for cancer therapeutics (Bai et al., 2019). However, whether C1QBP functions in TNBC cells under hypoxic conditions and mediates tumor-cell sensitivity to chemotherapy needs to be further investigated. Here, we report that, depletion of the expression of C1QBP induced by hypoxia significantly increased the sensitivity to PTX and reduced IC50 values (Figure 5B and Supplementary Figure 9B), suggesting that C1QBP is critical in mediating PTX resistance to TNBC cells. NF-κB signaling is activated and critical in modulating cell survival; it upregulates anti-apoptotic genes when cells are treated with chemotherapeutic drugs (Wu and Miyamoto, 2007; Baldwin, 2012). The PKC family of proteins comprises serine/threonine kinases (Hanks and Hunter, 1995) that are strongly associated with the chemoresistance of various tumors (Guha et al., 2010; Yun et al., 2010; Sreekumar et al., 2019). PKCs regulate the transcription of downstream target genes by activating NF-κB signaling (Diaz-Meco and Moscat, 2012). We have shown that hypoxia induces the expression of the majority of PKCs and NF-κB pathway family proteins; silencing C1QBP reversed the activation of these signaling pathways (Figure 5F). Moreover, we found out that MDR1, a PTX resistance-related protein (Wang et al., 2020), decreased in shC1QBP cells under hypoxic conditions (Supplementary Figure 9D). All these data suggest that C1QBP is important in the stimulation of the PKC–NF-κB–VCAM-1 pathway, which makes it a possible target for treating TNBC, especially in TNBC patients with low sensitivity to PTX or for treating/pre-treating patients to prevent metastasis. In addition, our data indicated that phosphorylated ser536 was upregulated after C1QBP knockdown in hypoxia (Figure 5G). It has been reported that phosphorylation of Ser536 in P65 inhibits intestinal, breast, and prostate cancers (Bu et al., 2016). Currently, 13 phosphorylation sites have been found in P65 as following: ser205, thr254, ser276, ser281, thr435, thr505, ser468, ser529, ser535, ser536, and ser537. Different phosphorylation sites may play diverse roles in tumor regulation. For instance, phosphorylation of ser276 promotes growth of head and neck squamous cell carcinoma (Arun et al., 2009), while thr-505 phosphorylation inhibits hepatoma cells (Moles et al., 2016). However, the mechanism of P65 phosphorylated at ser546 in TNBC cells under hypoxia needs further investigation.

There are still some open questions that need to be addressed. Firstly, the functions of C1QBP in other subtypes of breast cancer are still unclear and deserved to be explored. Secondly, studies have revealed that PKCs play an important role in increasing the transcription of HIF-1α (Page et al., 2002; Datta et al., 2004; Lee et al., 2007; Kim et al., 2016). The increase in mRNA and protein levels of HIF-1α were inhibited in C1QBP-depleted cells under hypoxia (Figure 5C); further experiments need to be carried out to determine whether there exists a C1QBP–PKC–HIF-1α regenerative feedback loop in TNBC under hypoxic conditions. Besides, changes in C1QBP mRNA and protein levels did not necessarily match (Figures 1C, 2A, 5D and Supplementary Figures 2E–G). Under hypoxic conditions, the activity of proline hydroxylase domain protein 2 (PHD2) is inhibited, leading to HIF-1α protein stabilization, DNA binding, coactivator recruitment, and target gene transactivation (Xiang et al., 2014). Under normal oxygen condition, the basic expression level of HIF-1α is low (Zhang et al., 2016). Besides, post-translational regulation of C1QBP protein level under hypoxic conditions may be responsible for the observed level of this protein. Supporting this hypothesis, it has been demonstrated that C1QBP protein interacts with PKCζ in MDA-MB-231 TNBC cells (Zhang et al., 2013). They also show that many proteins interact with C1QBP protein and involve in post-translational modification of C1QBP (such as Rnf2, psmd1, prkcz, prkci, etc.). These proteins regulate the process of post-translational modification, indicating that there exists post-translational modification of C1QBP protein in breast cancer. This might explain the reason why changes in C1QBP mRNA and protein levels did not necessarily match. Further analyses including Co-IP assay and /or proteomics should be performed to screen out interactive proteins (such as deubiquitinase) with C1QBP under hypoxia. Thirdly, it is well known that intratumoral hypoxia is a common characteristic of tumor microenvironment, including TNBC (Xiang et al., 2014). Accordingly, we suppose that hypoxic microenvironment was also established in our mice once the tumor was xenografted *in vivo*. However, it has been demonstrated that the mean partial pressure of oxygen (pO_2_) in breast tumors ranges from 2.5 to 28 mmHg, with a median value of 10 mmHg (1% O_2_), compared with 65 mmHg observed in normal human breast tissue (Vaupel et al., 2007). Supposedly, gene expression and functions (including C1QBP) may be affected by different pO_2_, although this hypothesis requires further testing. Future studies should explore tissue sections from different areas of the tumors with different levels of oxygenation *in vivo* and/or clinical tumor samples to further clarify the internal mechanisms. Finally, as C1QBP affected MDR1 protein expression under hypoxia (Supplementary Figure 9D), further investigations will be explored by using clinical samples and PTX-resistant TNBC cells to understand the potential correlations among C1QBP, P65 and MDR1. Organoids or PDX mouse models should be further explored to encapsulate silencing of the C1QBP gene vector by nanotechnology to verify its antitumor effect and assess its practical application value in clinical.

## Conclusion

We identified C1QBP as a novel mediator of hypoxia-related metastasis and chemoresistance in TNBC. In this study, we showed that hypoxia-induced HIF-1α promoted C1QBP upregulation upon transcriptional control, which may explain why this gene was overexpressed in TNBC. Silencing C1QBP downregulated the PKC-NF-κB-VCAM-1 signaling pathway and inhibited cell migration, invasion and PTX-resistant abilities under hypoxic conditions. Based on these findings, it can be inferred that this critical gene has potential as a therapeutic target in TNBC.

## Data Availability Statement

The datasets presented in this study can be found in online repositories. The names of the repository/repositories and accession number(s) can be found in the article/Supplementary Material.

## Ethics Statement

The studies involving human participants were reviewed and approved by the Harbin Medical University Cancer Hospital. The patients/participants provided their written informed consent to participate in this study. The animal study was reviewed and approved by the Harbin Medical University Cancer Hospital.

## Author Contributions

HW designed the experiments and wrote the manuscript. HW and YC carried out the experiments. HW, GL, YC, SX, YJ, XZ, SG, QW, and JZ analyzed the experimental results. HW, DP, SS, and SX funded the research. All authors contributed to the article and approved the submitted version.

## Conflict of Interest

The authors declare that the research was conducted in the absence of any commercial or financial relationships that could be construed as a potential conflict of interest.

## Footnotes

1 http://genome.ucsc.edu/

2 http://jaspar.genereg.net/

## Supplementary Material

Supplementary Figure 1 Hypoxia-induced HIF-1α promotes C1QBP upregulation in TNBC. **(A)** MDA-MB-468 cells were exposed to 20% or 1% O_2_ for 3, 6, 9, 12, 24, and 48 h. Western blot shows the protein levels of C1QBP at different points in time under normoxic and hypoxic conditions; β-actin levels were used as the internal control. **(B)** MDA-MB-468 cells were exposed to 20% or 1% O_2_ for 6 h; subsequently, total RNA was extracted and transcriptome analyses were performed for the two groups of treated cells. GO annotations for the up- and downregulated genes. **(C)** Enrichment of different mRNAs in the KEGG pathways. **(D)** Volcano plots show the different up- or downregulated transcripts under hypoxic conditions compared to those in a normoxic environment. **(E)** MDA-MB-468 cells were exposed to 20% or 1% O_2_ for 3, 6, 9, 12, 24, and 48 h. Real-time qPCR was performed to determine the mRNA levels of C1QBP; β-actin mRNA was the internal control. Western blot shows the protein levels of C1QBP at different points under normoxic and hypoxic conditions; β-actin levels were used as the internal control. Three independent experiments were used to represent the mean ± standard deviation values. *****P* < 0.0001.

Supplementary Figure 2 Hypoxia-induced HIF-1α promotes C1QBP upregulation in TNBC. **(A)** Cell viabilities of Hs578T or MDA-MB-468 were measured at 0, 24, 48, and 72 h upon treatment with shHIF-1α or HIF-1α inhibitor. **(B)** Western blot was performed to detect the expression of VEGF, VHL, E-cad and β-tubulin; β-actin levels were used as the internal control. **(C)** HIF-1α knockdown was confirmed using real-time qPCR and western blot after MDA-MB-468 cells were transfected with shRNA in a 20% O_2_ environment. **(D)** The expression of HIF-1α was confirmed using real-time qPCR after MDA-MB-468 cells were treated with the HIF-1α inhibitor (10 μmol/ml) in the presence of 20% O_2_ for 24 h. **(E)** The mRNA levels of C1QBP were quantified using real-time qPCR after transfected with shRNA. **(F)** The mRNA levels of C1QBP were detected using real-time qPCR after transfecting the cells with shHIF-1α. **(G)** Western blot was performed to detect the expression of C1QBP by different treatment; β-actin levels were used as the internal control. **(H)** Two of the potential binding sites on the promoter of C1QBP. **(I)** MDA-MB-468 cells were treated with the HIF-1α inhibitor (10 μmol/ml) under normoxic conditions for 24 h. ChIP was performed and binding at the two sites was quantified using real-time qPCR. **(J)** Comparison of the mRNA levels of C1QBP with HIF-1αexpression in breast cancer using data from the TCGA database. Three independent experiments were used to represent the mean ± standard deviation values. ***P* < 0.01 and ****P* < 0.0001.

Supplementary Figure 3 Knockdown of C1QBP inhibits TNBC migration, invasion and chemoresistance to paclitaxel *in vitro*. **(A)** The knockdown of C1QBP was confirmed using real-time qPCR and western blot after MDA-MB-468 cells were transfected with si3. **(B)** Migration and invasion analysis indicating differential cell motilities in MDA-MB-468 cells. **(C)** Cell viabilities of Hs578T were measured at 0, 24, 48, and 72 h upon treatment with si1, si2 or si3 under normoxia or hypoxia. **(D)** Cell viabilities of MDA-MB-468 were measured at 0, 24, 48, and 72 h upon treatment with shC1QBP under normoxia or hypoxia. Three independent experiments were used to represent the mean ± standard deviation values. **P* < 0.05 and ****P* < 0.0001.

Supplementary Figure 4 Knockdown of C1QBP hinders *in vivo* TNBC lung colonization and chemoresistance to paclitaxel. **(A)** Structure of lentiviral vectors containing GFP. **(B)** Lung colonization model. Representative mice were injected with 5 × 10^6^ cells. Luciferase signal intensity was measured on equivalent scales, with pre-treatment and post-treatment of saline or PTX. **(C)** Subcutaneous model. Representative mice were injected with 1 × 10^7^ cells. Luciferase signal intensity was measured on equivalent scales, with pre-treatment and post-treatment of saline or PTX.

Supplementary Figure 5 Knockdown of C1QBP hinders *in vivo* TNBC lung colonization and chemoresistance to paclitaxel. **(A)** HISTECH Pannoramic250 analysis of HE staining and tumors in the lung tissues of control or shC1QBP. **(B)** IHC staining shows the ki67 protein levels in the lung tissues. **(C)** IHC staining shows the ki67 protein levels in the subcutaneous tissues. Three independent experiments were used to represent the mean ± standard deviation values. **P* < 0.05, ***P* < 0.01, and ****P* < 0.0001.

Supplementary Figure 6 Depletion of C1QBP downregulates VCAM-1 expression in TNBC via P65 in the nucleus. **(A)** Volcano plots showing the up- or downregulated transcripts in the si1, si2, and si3 groups as compared to those in siNC. Hs578T cells were transfected with siRNA to silence the expression of C1QBP, and total RNA from the groups was extracted, following which, transcriptome analyses were performed. **(B)** Biological processes, cellular components, and molecular functions of the GO annotations of the genes. **(C)** The enrichment of various mRNAs in the KEGG pathways. **(D)** Volcano plots of the consistently identified up- and downregulated genes. **(E)** GO analysis of the consistently identified up- and downregulated genes.

Supplementary Figure 7 Depletion of C1QBP downregulates VCAM-1 expression in TNBC via P65 in the nucleus. **(A)** Real-time qPCR and western blot were used to determine the mRNA and protein levels of VCAM-1 after transfection with si3 in the MDA-MB-468 cells for 48 h. **(B)** The mRNA levels of α4, β1 and β7 in EA.hy926 cells were detected upon treatment with TNFα for 24 h using real-time qPCR. **(C)** EA.hy926 cells were treated with TNFα (1,000 U/ml) for 24 h. Hs578T or MDA-MB-468 (transfected with siNC and si3) cells (5 × 10^4^) were incubated with EA.hy926 cells in each well. Subsequently, 0.25% RB was added to each well and the absorbance was measured at 570 nm. **(D)** Structure of lentiviral vectors containing luciferase. **(E)** HUVECs were seeded in each well of the 96-well plate (2 × 10^4^ cells per well) and treated with TNFα (1,000 U/ml) for 24 h. MDA-MB-468-control and MDA-MB-468-shC1QBP cells (5 × 10^4^ cells/200 μl) were infected with the lentiviral vectors containing GFP and added onto HUVECs. The adherent TNBC cells were visualized and photomicrographs. The number of adherent TNBC cells with green fluorescence per field was calculated. Then RB was added per well and incubated. The number of RB-stained adherent cells was also calculated. **(F)** Comparison of the mRNA levels of C1QBP with VCAM-1 expression in breast cancer using data from the TCGA database. Three independent experiments were used to represent the mean ± standard deviation values. **P* < 0.05, ***P* < 0.01, and ****P* < 0.0001.

Supplementary Figure 8 Depletion of C1QBP downregulates VCAM-1 expression in TNBC via P65 in the nucleus. **(A)** The NF-κB pathway was shown to be enriched by KEGG analysis after treating the Hs578T cells with siC1QBP. Green boxes represent downregulated genes; VCAM-1 belongs to the NF-κB pathway. **(B)** The mRNA levels of TNF were detected in control and shC1QBP cells by real-time qPCR under normoxia. **(C)** IHC staining shows the VCAM-1 protein levels in the subcutaneous tissues. **(D)** IHC staining shows the VCAM-1 protein levels in the lung tissues. Three independent experiments were used to represent the mean ± standard deviation values. **P* < 0.05 and *****P* < 0.0001.

Supplementary Figure 9 Depletion of C1QBP inhibits hypoxia-induced activation of the PKC–NF-κB–VCAM-1 signaling pathway. **(A)** Hs578T cells transfecting with siNC, si1, si2, and si3 were exposed to 1% O_2_ for 48 h and the potential for migration and invasion were measured using transwell assay. **(B)** Hs578T cells transfecting with siNC and si3 were incubated with PTX under exposure to 1% O_2_ for 24 h. The upper panel shows cell viabilities using MTS assay. The lower panel shows the comparison of the IC50 value and resistance index of siNC and si3 samples. **(C)** The mRNA levels of TNF were detected in control and shC1QBP cells by real-time qPCR under hypoxia. **(D)** The protein levels of MDR1 were detected in control and shC1QBP cells by western blot under hypoxia. **(E)** MDA-MB-468 control and shC1QBP cells were transfected with MDR1-overexpression plasmid under hypoxia. The protein levels of MDR1 and C1QBP were detected by western blot. **(F)** MDA-MB-468 control and shC1QBP cells transfecting with MDR1-overexpression plasmid were incubated with PTX under exposure to 1% O_2_ for 24 h. The upper panel shows cell viabilities using MTS assay. The lower panel shows the comparison of the IC50 values and resistance index of each group. Three independent experiments were used to represent the mean ± standard deviation values. ^∗∗^*P* < 0.01, ^∗∗∗^*P* < 0.0001, and ^****^*P* < 0.0001.

Supplementary Figure 10 The C1QBP level correlates with VCAM-1 and P65 expression in TNBC patient tissues. **(A)** Correlation analyses of the IHC staining scores of C1QBP and VCAM-1 levels in the tumor cells of all patients with breast cancer. **(B)** Correlation analyses of the IHC staining scores of C1QBP and P65 levels in the tumor cells of all patients with breast cancer. **(C)** Correlation analyses of the IHC staining scores of C1QBP and HIF-1α levels in the tumor cells of all patients with breast cancer. **(D)** The correlation analyses of the IHC staining scores of the HIF-1α and VCAM-1 levels in the tumor cells of breast cancer patients. **(E)** The correlation analyses of the IHC staining scores of the HIF-1α and P65 levels in the tumor cells of all breast cancer patients. **(F)** The correlation analyses of the IHC staining scores of the P65 and VCAM-1 levels in the tumor cells of all breast cancer patients. **(G)** The correlation analyses of the IHC staining scores of the HIF-1α and VCAM-1 levels in the tumor cells of TNBC patients. **(H)** The correlation analyses of the IHC staining scores of the HIF-1α and P65 levels in the tumor cells of TNBC patients. **(I)** The correlation analyses of the IHC staining scores of the P65 and VCAM-1 levels in the tumor cells of TNBC patients.

## Abbreviations

C1QBP: complement 1q binding protein
TNBC: triple-negative breast cancer
PTX: paclitaxel
HIF-1 α: hypoxia inducible factor 1 subunit alpha
VCAM-1: vascular cell adhesion molecule 1
PKC: protein kinase C
TNF α: tumor necrosis factor α
NF- κ B: nuclear factor-kappa B
P65: RELA proto-oncogene nuclear factor-kappa B subunit
PHD2: proline hydroxylase domain protein 2
I κ B α: nuclear factor-kappa B inhibitor alpha
VEGF: vascular endothelial growth factor
VHL: von Hippel-Lindau tumor suppressor
E-cad: E-cadherin 1
TNF: tumor necrosis factor
MDR1: ATP binding cassette subfamily B member 1
TP53INP1: tumor protein p53 inducible nuclear protein 1
ChIP: chromatin immunoprecipitation
TCGA: The Cancer Genome Atlas
GO: gene ontology
KEGG: Kyoto Encyclopedia of Genes and Genomes
IHC: immunohistochemistry
HE: hematoxylin-eosin.

## References

[B1] Arun P. Brown M. S. Ehsanian R. Chen Z. Van Waes C. (2009). Nuclear NF-kappaB p65 phosphorylation at serine 276 by protein kinase A contributes to the malignant phenotype of head and neck cancer. *Clin. Cancer Res*. 15 5974–5984. 10.1158/1078-0432.CCR-09-1352 19789307 PMC2760015

[B2] Bai Y. Wang W. Li S. Zhan J. Li H. Zhao M. (2019). C1QBP Promotes Homologous Recombination by Stabilizing MRE11 and Controlling the Assembly and Activation of MRE11/RAD50/NBS1 Complex. *Mol. Cell*. 75 1299–1314 e6. 10.1016/j.molcel.2019.06.023 31353207

[B3] Baldwin A. S. (2012). Regulation of cell death and autophagy by IKK and NF-kappaB: critical mechanisms in immune function and cancer. *Immunol. Rev*. 246 327–345. 10.1111/j.1600-065X.2012.01095.x 22435564

[B4] Bordji K. Grandval A. Cuhna-Alves L. Lechapt-Zalcman E. Bernaudin M. (2014). Hypoxia-inducible factor-2alpha (HIF-2alpha), but not HIF-1alpha, is essential for hypoxic induction of class III beta-tubulin expression in human glioblastoma cells. *FEBS J*. 281 5220–5236. 10.1111/febs.13062 25244496

[B5] Bray F. Ferlay J. Soerjomataram I. Siegel R. L. Torre L. A. Jemal A. (2018). Global cancer statistics 2018: GLOBOCAN estimates of incidence and mortality worldwide for 36 cancers in 185 countries. *CA Cancer J. Clin*. 68 394–424. 10.3322/caac.21492 30207593

[B6] Brezinschek R. I. Brezinschek H. P. Lazarovits A. I. Lipsky P. E. Oppenheimer-Marks N. (1996). Expression of the beta 7 integrin by human endothelial cells. *Am. J. Pathol*. 149 1651–1660. 8909254 PMC1865263

[B7] Bu Y. Li X. He Y. Huang C. Shen Y. Cao Y. (2016). A phosphomimetic mutant of RelA/p65 at Ser536 induces apoptosis and senescence: An implication for tumor-suppressive role of Ser536 phosphorylation. *Int. J. Cancer* 138 1186–1198. 10.1002/ijc.29852 26375985

[B8] Cai H. Wang C. Shukla S. Steinmetz N. F. (2019). Cowpea Mosaic Virus Immunotherapy Combined with Cyclophosphamide Reduces Breast Cancer Tumor Burden and Inhibits Lung Metastasis. *Adv. Sci.* 6:1802281. 10.1002/advs.201802281 31453050 PMC6702650

[B9] Cao S. Li Q. Hou J. Li Z. Cao X. Liu X. (2019). Intrathecal TRPM8 blocking attenuates cold hyperalgesia via PKC and NF-kappaB signaling in the dorsal root ganglion of rats with neuropathic pain. *J. Pain Res*. 12 1287–1296. 10.2147/JPR.S197168 31114308 PMC6497852

[B10] Cartee T. V. White K. J. Newton-West M. Swerlick R. A. (2012). Hypoxia and hypoxia mimetics inhibit TNF-dependent VCAM1 induction in the 5A32 endothelial cell line via a hypoxia inducible factor dependent mechanism. *J. Dermatol. Sci*. 65 86–94. 10.1016/j.jdermsci.2011.10.003 22093255 PMC3273579

[B11] Chaffer C. L. Weinberg R. A. (2011). A perspective on cancer cell metastasis. *Science* 331 1559–1564. 10.1126/science.1203543 21436443

[B12] Chen C. Zhang Q. Liu S. Parajuli K. R. Qu Y. Mei J. (2015). IL-17 and insulin/IGF1 enhance adhesion of prostate cancer cells to vascular endothelial cells through CD44-VCAM-1 interaction. *Prostate* 75 883–895. 10.1002/pros.22971 25683512 PMC4405436

[B13] Chen J. Miao W. Yang S. Yin M. Zhao J. Song D. (2020). LncRNA NR_027471 Functions as a ceRNA for miRNA-8055 Leading to Suppression of Osteosarcoma by Regulating the Expression of TP53INP1. *Front. Oncol*. 10:563255. 10.3389/fonc.2020.563255 33117693 PMC7550745

[B14] Chen Q. Massague J. (2012). Molecular pathways: VCAM-1 as a potential therapeutic target in metastasis. *Clin. Cancer Res*. 18 5520–5525. 10.1158/1078-0432.CCR-11-2904 22879387 PMC3473104

[B15] Chen Y. B. Jiang C. T. Zhang G. Q. Wang J. S. Pang D. (2009). Increased expression of hyaluronic acid binding protein 1 is correlated with poor prognosis in patients with breast cancer. *J. Surg. Oncol*. 100 382–386. 10.1002/jso.21329 19565630

[B16] Clevers H. Nusse R. (2012). Wnt/beta-catenin signaling and disease. *Cell* 149 1192–1205. 10.1016/j.cell.2012.05.012 22682243

[B17] Cruz F. Vilela R. A. Ferreira E. B. Melo N. S. Reis P. (2019). Evidence on the Use of Mobile Apps During the Treatment of Breast Cancer: Systematic Review. *JMIR Mhealth Uhealth* 7:e13245. 10.2196/13245 31456578 PMC6734853

[B18] Dan Z. Cao H. He X. Zhang Z. Zou L. Zeng L. (2016). A pH-Responsive Host-guest Nanosystem Loading Succinobucol Suppresses Lung Metastasis of Breast Cancer. *Theranostics* 6 435–445. 10.7150/thno.13896 26909117 PMC4737729

[B19] Datta K. Li J. Bhattacharya R. Gasparian L. Wang E. Mukhopadhyay D. (2004). Protein kinase C zeta transactivates hypoxia-inducible factor alpha by promoting its association with p300 in renal cancer. *Cancer Res*. 64 456–462. 10.1158/0008-5472.can-03-2706 14744756

[B20] Diaz-Meco M. T. Moscat J. (2012). The atypical PKCs in inflammation: NF-kappaB and beyond. *Immunol. Rev*. 246 154–167. 10.1111/j.1600-065X.2012.01093.x 22435553 PMC3531713

[B21] Fan G. H. Zhu T. Y. Huang J. (2020). FNDC5 promotes paclitaxel sensitivity of non-small cell lung cancers via inhibiting MDR1. *Cell Signal* 72:109665. 10.1016/j.cellsig.2020.109665 32353410

[B22] Feichtinger R. G. Olahova M. Kishita Y. Garone C. Kremer L. S. Yagi M. (2017). Biallelic C1QBP Mutations Cause Severe Neonatal-, Childhood-, or Later-Onset Cardiomyopathy Associated with Combined Respiratory-Chain Deficiencies. *Am. J. Hum. Genet*. 101 525–538. 10.1016/j.ajhg.2017.08.015 28942965 PMC5630164

[B23] Fogal V. Babic I. Chao Y. Pastorino S. Mukthavaram R. Jiang P. (2015). Mitochondrial p32 is upregulated in Myc expressing brain cancers and mediates glutamine addiction. *Oncotarget* 6 1157–1170. 10.18632/oncotarget.2708 25528767 PMC4359224

[B24] Fogal V. Richardson A. D. Karmali P. P. Scheffler I. E. Smith J. W. Ruoslahti E. (2010). Mitochondrial p32 protein is a critical regulator of tumor metabolism via maintenance of oxidative phosphorylation. *Mol. Cell Biol*. 30 1303–1318. 10.1128/MCB.01101-09 20100866 PMC2832503

[B25] Fogal V. Zhang L. Krajewski S. Ruoslahti E. (2008). Mitochondrial/cell-surface protein p32/gC1qR as a molecular target in tumor cells and tumor stroma. *Cancer Res*. 68 7210–7218. 10.1158/0008-5472.CAN-07-6752 18757437 PMC2562323

[B26] Gamble J. R. Vadas M. A. (1988). A new assay for the measurement of the attachment of neutrophils and other cell types to endothelial cells. *J. Immunol. Methods* 109 175–184. 10.1016/0022-1759(88)90240-2 3361131

[B27] Garmy-Susini B. Avraamides C. J. Desgrosellier J. S. Schmid M. C. Foubert P. Ellies L. G. (2013). PI3Kalpha activates integrin alpha4beta1 to establish a metastatic niche in lymph nodes. *Proc. Natl. Acad. Sci. U. S. A*. 110 9042–9047. 10.1073/pnas.1219603110 23671068 PMC3670313

[B28] Gilkes D. M. Semenza G. L. Wirtz D. (2014). Hypoxia and the extracellular matrix: drivers of tumour metastasis. *Nat. Rev. Cancer* 14 430–439. 10.1038/nrc3726 24827502 PMC4283800

[B29] Giridharan S. Srinivasan M. (2018). Mechanisms of NF-kappaB p65 and strategies for therapeutic manipulation. *J. Inflamm. Res*. 11 407–419. 10.2147/JIR.S140188 30464573 PMC6217131

[B30] Guan Z. Ding C. Du Y. Zhang K. Zhu J. N. Zhang T. (2014). HAF drives the switch of HIF-1alpha to HIF-2alpha by activating the NF-kappaB pathway, leading to malignant behavior of T24 bladder cancer cells. *Int. J. Oncol*. 44 393–402. 10.3892/ijo.2013.2210 24316875 PMC3898811

[B31] Guha S. Tanasanvimon S. Sinnett-Smith J. Rozengurt E. (2010). Role of protein kinase D signaling in pancreatic cancer. *Biochem. Pharmacol*. 80 1946–1954. 10.1016/j.bcp.2010.07.002 20621068 PMC2974013

[B32] Hanks S. K. Hunter T. (1995). Protein kinases 6. The eukaryotic protein kinase superfamily: kinase (catalytic) domain structure and classification. *FASEB J*. 9 576–596. 10.1096/fasebj.9.8.7768349 7768349

[B33] Ibuki M. Lee D. Shinojima A. Miwa Y. Tsubota K. Kurihara T. (2020). Rice Bran and Vitamin B6 Suppress Pathological Neovascularization in a Murine Model of Age-Related Macular Degeneration as Novel HIF Inhibitors. *Int. J. Mol. Sci*. 21:940. 10.3390/ijms21238940 33255657 PMC7728083

[B34] Ireson C. R. Alavijeh M. S. Palmer A. M. Fowler E. R. Jones H. J. (2019). The role of mouse tumour models in the discovery and development of anticancer drugs. *Br. J. Cancer* 121 101–108. 10.1038/s41416-019-0495-5 31231121 PMC6738037

[B35] Isakov N. (2018). Protein kinase C (PKC) isoforms in cancer, tumor promotion and tumor suppression. *Semin. Cancer Biol*. 48 36–52. 10.1016/j.semcancer.2017.04.012 28571764

[B36] Jyotsana N. Zhang Z. Himmel L. E. Yu F. King M. R. (2019). Minimal dosing of leukocyte targeting TRAIL decreases triple-negative breast cancer metastasis following tumor resection. *Sci. Adv*. 5:eaaw4197. 10.1126/sciadv.aaw4197 31355333 PMC6656540

[B37] Keith B. Johnson R. S. Simon M. C. (2011). HIF1alpha and HIF2alpha: sibling rivalry in hypoxic tumour growth and progression. *Nat. Rev. Cancer* 12 9–22. 10.1038/nrc3183 22169972 PMC3401912

[B38] Khan M. A. Jain V. K. Rizwanullah M. Ahmad J. Jain K. (2019). PI3K/AKT/mTOR pathway inhibitors in triple-negative breast cancer: a review on drug discovery and future challenges. *Drug Discov. Today* 24 2181–2191. 10.1016/j.drudis.2019.09.001 31520748

[B39] Kim H. Na Y. R. Kim S. Y. Yang E. G. (2016). Protein Kinase C Isoforms Differentially Regulate Hypoxia-Inducible Factor-1alpha Accumulation in Cancer Cells. *J. Cell. Biochem*. 117 647–658. 10.1002/jcb.25314 26284819

[B40] Kim K. B. Yi J. S. Nguyen N. Lee J. H. Kwon Y. C. Ahn B. Y. (2011). Cell-surface receptor for complement component C1q (gC1qR) is a key regulator for lamellipodia formation and cancer metastasis. *J. Biol. Chem*. 286 23093–23101. 10.1074/jbc.M111.233304 21536672 PMC3123076

[B41] Labrousse-Arias D. Martinez-Alonso E. Corral-Escariz M. Bienes-Martinez R. Berridy J. Serrano-Oviedo L. (2017). VHL promotes immune response against renal cell carcinoma via NF-kappaB-dependent regulation of VCAM-1. *J. Cell. Biol*. 216 835–847. 10.1083/jcb.201608024 28235946 PMC5350518

[B42] Lee J. W. Park J. A. Kim S. H. Seo J. H. Lim K. J. Jeong J. W. (2007). Protein kinase C-delta regulates the stability of hypoxia-inducible factor-1 alpha under hypoxia. *Cancer Sci*. 98 1476–1481. 10.1111/j.1349-7006.2007.00535.x 17608772 PMC11160108

[B43] Lee K. M. Guerrero-Zotano A. L. Servetto A. Sudhan D. R. Lin C. C. Formisano L. (2020). Proline rich 11 (PRR11) overexpression amplifies PI3K signaling and promotes antiestrogen resistance in breast cancer. *Nat. Commun*. 11:5488. 10.1038/s41467-020-19291-x 33127913 PMC7599336

[B44] Lei B. Wang D. Zhang M. Deng Y. Jiang H. Li Y. (2020). miR-615-3p promotes the epithelial-mesenchymal transition and metastasis of breast cancer by targeting PICK1/TGFBRI axis. *J. Exp. Clin. Cancer Res*. 39:71. 10.1186/s13046-020-01571-5 32336285 PMC7183699

[B45] Li N. Cui T. Guo W. Wang D. Mao L. (2019). MiR-155-5p accelerates the metastasis of cervical cancer cell via targeting TP53INP1. *Onco. Targets Ther*. 12 3181–3196. 10.2147/OTT.S193097 31118671 PMC6500876

[B46] Li W. Zhang X. Wang W. Sun R. Liu B. Ma Y. (2017). Quantitative proteomics analysis of mitochondrial proteins in lung adenocarcinomas and normal lung tissue using iTRAQ and tandem mass spectrometry. *Am. J. Transl. Res*. 9 3918–3934. 28979670 PMC5622239

[B47] Li Y. Luan C. (2018). PLCE1 Promotes the Invasion and Migration of Esophageal Cancer Cells by Up-Regulating the PKCalpha/NF-kappaB Pathway. *Yonsei. Med. J*. 59 1159–1165. 10.3349/ymj.2018.59.10.1159 30450849 PMC6240569

[B48] Liang X. Arullampalam P. Yang Z. Ming X. F. (2019). Hypoxia Enhances Endothelial Intercellular Adhesion Molecule 1 Protein Level Through Upregulation of Arginase Type II and Mitochondrial Oxidative Stress. *Front. Physiol*. 10:1003. 10.3389/fphys.2019.01003 31474872 PMC6702258

[B49] Lin C. C. Pan C. S. Wang C. Y. Liu S. W. Hsiao L. D. Yang C. M. (2015). Tumor necrosis factor-alpha induces VCAM-1-mediated inflammation via c-Src-dependent transactivation of EGF receptors in human cardiac fibroblasts. *J. Biomed. Sci*. 22:53. 10.1186/s12929-015-0165-8 26173590 PMC4502472

[B50] Liu T. Sun H. Zhu D. Dong X. Liu F. Liang X. (2017). TRA2A Promoted Paclitaxel Resistance and Tumor Progression in Triple-Negative Breast Cancers via Regulating Alternative Splicing. *Mol. Cancer Ther*. 16 1377–1388. 10.1158/1535-7163.MCT-17-0026 28416606

[B51] Liu Z. J. Semenza G. L. Zhang H. F. (2015). Hypoxia-inducible factor 1 and breast cancer metastasis. *J. Zhejiang Univ. Sci. B*. 16 32–43. 10.1631/jzus.B1400221 25559953 PMC4288942

[B52] Lu H. Samanta D. Xiang L. Zhang H. Hu H. Chen I. (2015). Chemotherapy triggers HIF-1-dependent glutathione synthesis and copper chelation that induces the breast cancer stem cell phenotype. *Proc. Natl. Acad. Sci. U. S. A*. 112 E4600–E4609. 10.1073/pnas.1513433112 26229077 PMC4547233

[B53] Masuelli L. Granato M. Benvenuto M. Mattera R. Bernardini R. Mattei M. (2017). Chloroquine supplementation increases the cytotoxic effect of curcumin against Her2/neu overexpressing breast cancer cells *in vitro* and *in vivo* in nude mice while counteracts it in immune competent mice. *Oncoimmunology* 6:e1356151. 10.1080/2162402X.2017.1356151 29147611 PMC5674961

[B54] McGee A. M. Baines C. P. (2011). Complement 1q-binding protein inhibits the mitochondrial permeability transition pore and protects against oxidative stress-induced death. *Biochem. J*. 433 119–125. 10.1042/BJ20101431 20950273 PMC3512559

[B55] Moles A. Butterworth J. A. Sanchez A. Hunter J. E. Leslie J. Sellier H. (2016). A RelA(p65) Thr505 phospho-site mutation reveals an important mechanism regulating NF-kappaB-dependent liver regeneration and cancer. *Oncogene* 35 4623–4632. 10.1038/onc.2015.526 26853469 PMC4862573

[B56] Ng K. Y. Chan L. H. Chai S. Tong M. Guan X. Y. Lee N. P. (2017). TP53INP1 Downregulation Activates a p73-Dependent DUSP10/ERK Signaling Pathway to Promote Metastasis of Hepatocellular Carcinoma. *Cancer Res*. 77 4602–4612. 10.1158/0008-5472.CAN-16-3456 28674078

[B57] Ning X. Wang Y. Yan W. Li G. Sang N. (2018). Chitin synthesis inhibitors promote liver cancer cell metastasis via interfering with hypoxia-inducible factor 1alpha. *Chemosphere* 206 231–237. 10.1016/j.chemosphere.2018.05.014 29753285

[B58] Niu M. Sun S. Zhang G. Zhao Y. Pang D. Chen Y. (2015). Elevated expression of HABP1 is correlated with metastasis and poor survival in breast cancer patients. *Am. J. Cancer Res*. 5 1190–1198. 26045997 PMC4449446

[B59] Oeckinghaus A. Ghosh S. (2009). The NF-kappaB family of transcription factors and its regulation. *Cold Spring Harb. Perspect. Biol*. 1:a000034. 10.1101/cshperspect.a000034 20066092 PMC2773619

[B60] Ozpolat B. Akar U. Mehta K. Lopez-Berestein G. (2007). PKC delta and tissue transglutaminase are novel inhibitors of autophagy in pancreatic cancer cells. *Autophagy* 3 480–483. 10.4161/auto.4349 17507797

[B61] Page E. L. Robitaille G. A. Pouyssegur J. Richard D. E. (2002). Induction of hypoxia-inducible factor-1alpha by transcriptional and translational mechanisms. *J. Biol. Chem*. 277 48403–48409. 10.1074/jbc.M209114200 12379645

[B62] Qureshi-Baig K. Kuhn D. Viry E. Pozdeev V. I. Schmitz M. Rodriguez F. (2019). Hypoxia-induced autophagy drives colorectal cancer initiation and progression by activating the PRKC/PKC-EZR (ezrin) pathway. *Autophagy* 27 1–17. 10.1080/15548627.2019.1687213 31775562 PMC7469473

[B63] Saha P. Ghosh I. Datta K. (2014). Increased hyaluronan levels in HABP1/p32/gC1qR overexpressing HepG2 cells inhibit autophagic vacuolation regulating tumor potency. *PLoS One*. 9:e103208. 10.1371/journal.pone.0103208 25061661 PMC4111551

[B64] Semenza G. L. (2012). Hypoxia-inducible factors in physiology and medicine. *Cell* 148 399–408. 10.1016/j.cell.2012.01.021 22304911 PMC3437543

[B65] Seux M. Peuget S. Montero M. P. Siret C. Rigot V. Clerc P. (2011). TP53INP1 decreases pancreatic cancer cell migration by regulating SPARC expression. *Oncogene* 30 3049–3061. 10.1038/onc.2011.25 21339733

[B66] Sharma P. (2018). Update on the Treatment of Early-Stage Triple-Negative Breast Cancer. *Curr. Treat Options Oncol*. 19:22. 10.1007/s11864-018-0539-8 29656345

[B67] Shetti D. Zhang B. Fan C. Mo C. Lee B. H. Wei K. (2019). Low Dose of Paclitaxel Combined with XAV939 Attenuates Metastasis, Angiogenesis and Growth in Breast Cancer by Suppressing Wnt Signaling. *Cells* 8:892. 10.3390/cells8080892 31416135 PMC6721645

[B68] Shi H. Fang W. Liu M. Fu D. (2017). Complement component 1, q subcomponent binding protein (C1QBP) in lipid rafts mediates hepatic metastasis of pancreatic cancer by regulating IGF-1/IGF-1R signaling. *Int. J. Cancer* 141 1389–1401. 10.1002/ijc.30831 28608366

[B69] Shokeen M. Zheleznyak A. Wilson J. M. Jiang M. Liu R. Ferdani R. (2012). Molecular imaging of very late antigen-4 (alpha4beta1 integrin) in the premetastatic niche. *J. Nucl. Med*. 53 779–786. 10.2967/jnumed.111.100073 22496586 PMC8522701

[B70] Sreekumar R. Emaduddin M. Al-Saihati H. Moutasim K. Chan J. Spampinato M. (2019). Protein kinase C inhibitors override ZEB1-induced chemoresistance in HCC. *Cell Death Dis*. 10:703. 10.1038/s41419-019-1885-6 31543517 PMC6755133

[B71] Urrutia A. A. Guan N. Mesa-Ciller C. Afzal A. Davidoff O. Haase V. H. (2020). Inactivation of HIF-prolyl 4-hydroxylases 1, 2 and 3 in NG2-expressing cells induces HIF2-mediated neurovascular expansion independent of erythropoietin. *Acta Physiol.* 47:e13547. 10.1111/apha.13547 32846048 PMC7757172

[B72] Vaupel P. Hockel M. Mayer A. (2007). Detection and characterization of tumor hypoxia using pO2 histography. *Antioxid Redox Signal*. 9 1221–1235. 10.1089/ars.2007.1628 17536958

[B73] Voloshin T. Gingis-Velitski S. Bril R. Benayoun L. Munster M. Milsom C. (2011). G-CSF supplementation with chemotherapy can promote revascularization and subsequent tumor regrowth: prevention by a CXCR4 antagonist. *Blood* 118 3426–3435. 10.1182/blood-2010-11-320812 21685373 PMC3179406

[B74] Wang C. Guan W. Peng J. Chen Y. Xu G. Dou H. (2020). Gene/paclitaxel co-delivering nanocarriers prepared by framework-induced self-assembly for the inhibition of highly drug-resistant tumors. *Acta Biomater*. 103 247–258. 10.1016/j.actbio.2019.12.015 31846802

[B75] Wang G. L. Jiang B. H. Rue E. A. Semenza G. L. (1995). Hypoxia-inducible factor 1 is a basic-helix-loop-helix-PAS heterodimer regulated by cellular O2 tension. *Proc. Natl. Acad. Sci. U. S. A*. 92 5510–5514. 10.1073/pnas.92.12.5510 7539918 PMC41725

[B76] Wang J. Song Y. Liu T. Shi Q. Zhong Z. Wei W. (2015). Elevated expression of HABP1 is a novel prognostic indicator in triple-negative breast cancers. *Tumour. Biol*. 36 4793–4799. 10.1007/s13277-015-3131-x 25794640

[B77] Wei Q. Li Y. X. Liu M. Li X. Tang H. (2012). MiR-17-5p targets TP53INP1 and regulates cell proliferation and apoptosis of cervical cancer cells. *IUBMB Life* 64 697–704. 10.1002/iub.1051 22730212

[B78] Wu Q. Zhou W. Yin S. Zhou Y. Chen T. Qian J. (2019). Blocking Triggering Receptor Expressed on Myeloid Cells-1-Positive Tumor-Associated Macrophages Induced by Hypoxia Reverses Immunosuppression and Anti-Programmed Cell Death Ligand 1 Resistance in Liver Cancer. *Hepatology* 70 198–214. 10.1002/hep.30593 30810243 PMC6618281

[B79] Wu Z. Zuo M. Zeng L. Cui K. Liu B. Yan C. (2020). OMA1 reprograms metabolism under hypoxia to promote colorectal cancer development. *EMBO Rep.* 27:e50827. 10.15252/embr.202050827 33314701 PMC7788456

[B80] Wu Z. H. Miyamoto S. (2007). Many faces of NF-kappaB signaling induced by genotoxic stress. *J. Mol. Med.* 85 1187–1202. 10.1007/s00109-007-0227-9 17607554

[B81] Xiang L. Gilkes D. M. Chaturvedi P. Luo W. Hu H. Takano N. (2014). Ganetespib blocks HIF-1 activity and inhibits tumor growth, vascularization, stem cell maintenance, invasion, and metastasis in orthotopic mouse models of triple-negative breast cancer. *J. Mol. Med.* 92 151–164. 10.1007/s00109-013-1102-5 24248265 PMC3946681

[B82] Xie Z. B. Yao L. Jin C. Zhang Y. F. Fu D. L. (2019). High cytoplasm HABP1 expression as a predictor of poor survival and late tumor stage in pancreatic ductal adenocarcinoma patients. *Eur. J. Surg. Oncol*. 45 207–212. 10.1016/j.ejso.2018.09.020 30389300

[B83] Xu S. Wang P. Zhang J. Wu H. Sui S. Zhang J. (2019). Ai-lncRNA EGOT enhancing autophagy sensitizes paclitaxel cytotoxicity via upregulation of ITPR1 expression by RNA-RNA and RNA-protein interactions in human cancer. *Mol. Cancer* 18:89. 10.1186/s12943-019-1017-z 30999914 PMC6471868

[B84] Yan Y. Liu F. Han L. Zhao L. Chen J. Olopade O. I. (2018). HIF-2alpha promotes conversion to a stem cell phenotype and induces chemoresistance in breast cancer cells by activating Wnt and Notch pathways. *J. Exp. Clin. Cancer Res*. 37:256. 10.1186/s13046-018-0925-x 30340507 PMC6194720

[B85] Yang M. Zhu M. Song K. Wuren T. Yan J. Ge R. L. (2020). VHL gene methylation contributes to excessive erythrocytosis in chronic mountain sickness rat model by upregulating the HIF-2alpha/EPO pathway. *Life Sci*. 266:118873. 10.1016/j.lfs.2020.118873 33309718

[B86] Yu F. Wang L. Zhang B. (2019). Long non-coding RNA DRHC inhibits the proliferation of cancer cells in triple negative breast cancer by downregulating long non-coding RNA HOTAIR. *Oncol. Lett*. 18 3817–3822. 10.3892/ol.2019.10683 31516593 PMC6733000

[B87] Yun B. R. Lee M. J. Kim J. H. Kim I. H. Yu G. R. Kim D. G. (2010). Enhancement of parthenolide-induced apoptosis by a PKC-alpha inhibition through heme oxygenase-1 blockage in cholangiocarcinoma cells. *Exp. Mol. Med*. 42 787–797. 10.3858/emm.2010.42.11.082 20938215 PMC2992858

[B88] Yun J. Hong M. H. Kim S. Y. Park C. W. Kim S. Yun M. R. (2019). YH25448, an Irreversible EGFR-TKI with Potent Intracranial Activity in EGFR Mutant Non-Small Cell Lung Cancer. *Clin. Cancer Res*. 25 2575–2587. 10.1158/1078-0432.CCR-18-2906 30670498

[B89] Zerfaoui M. Suzuki Y. Naura A. S. Hans C. P. Nichols C. Boulares A. H. (2008). Nuclear translocation of p65 NF-kappaB is sufficient for VCAM-1, but not ICAM-1, expression in TNF-stimulated smooth muscle cells: Differential requirement for PARP-1 expression and interaction. *Cell Signal*. 20 186–194. 10.1016/j.cellsig.2007.10.007 17993261 PMC2278030

[B90] Zhang C. Samanta D. Lu H. Bullen J. W. Zhang H. Chen I. (2016). Hypoxia induces the breast cancer stem cell phenotype by HIF-dependent and ALKBH5-mediated m(6)A-demethylation of NANOG mRNA. *Proc. Natl. Acad. Sci. U. S. A*. 113 E2047–E2056. 10.1073/pnas.1602883113 27001847 PMC4833258

[B91] Zhang X. Zhang F. Guo L. Wang Y. Zhang P. Wang R. (2013). Interactome analysis reveals that C1QBP (complement component 1, q subcomponent binding protein) is associated with cancer cell chemotaxis and metastasis. *Mol. Cell Proteomics* 12 3199–3209. 10.1074/mcp.M113.029413 23924515 PMC3820933

[B92] Zhou X. Wu X. Qin L. Lu S. Zhang H. Wei J. (2020). Anti-Breast Cancer Effect of 2-Dodecyl-6-Methoxycyclohexa-2,5-Diene-1,4-Dione in vivo and in vitro Through MAPK Signaling Pathway. *Drug. Des. Devel. Ther*. 14 2667–2684. 10.2147/DDDT.S237699 32764871 PMC7369253

