# Supplementary figures and images for: Hypoxia-Mediated Complement 1q Binding Protein Regulates Metastasis and Chemoresistance in Triple-Negative Breast Cancer and Modulates the PKC-NF-κB-VCAM-1 Signaling Pathway
Source: Front Cell Dev Biol. 2021 Feb 23;9:607142. doi: 10.3389/fcell.2021.607142 (PMC7940382; doi:10.3389/fcell.2021.607142)

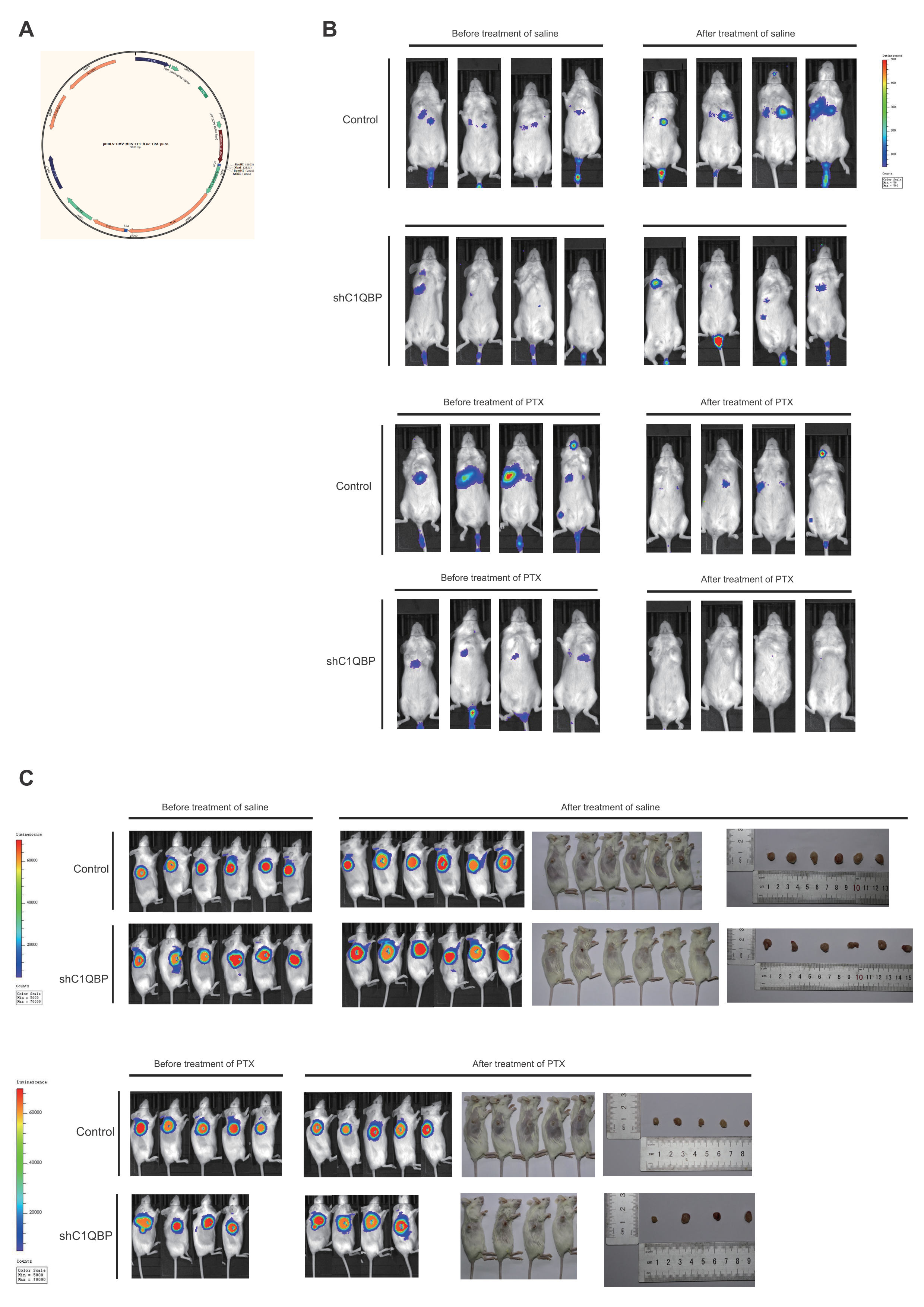

Supplement: Supplementary Figure 4 — Knockdown of C1QBP hinders in vivo TNBC lung colonization and chemoresistance to paclitaxel. (A) Structure of lentiviral vectors containing GFP. (B) Lung colonization model. Representative mice were injected with 5 × 10^6^ cells. Luciferase signal intensity was measured on equivalent scales, with pre-treatment and post-treatment of saline or PTX. (C) Subcutaneous model. Representative mice were injected with 1 × 10^7^ cells. Luciferase signal intensity was measured on equivalent scales, with pre-treatment and post-treatment of saline or PTX. [file Image_4.TIF]

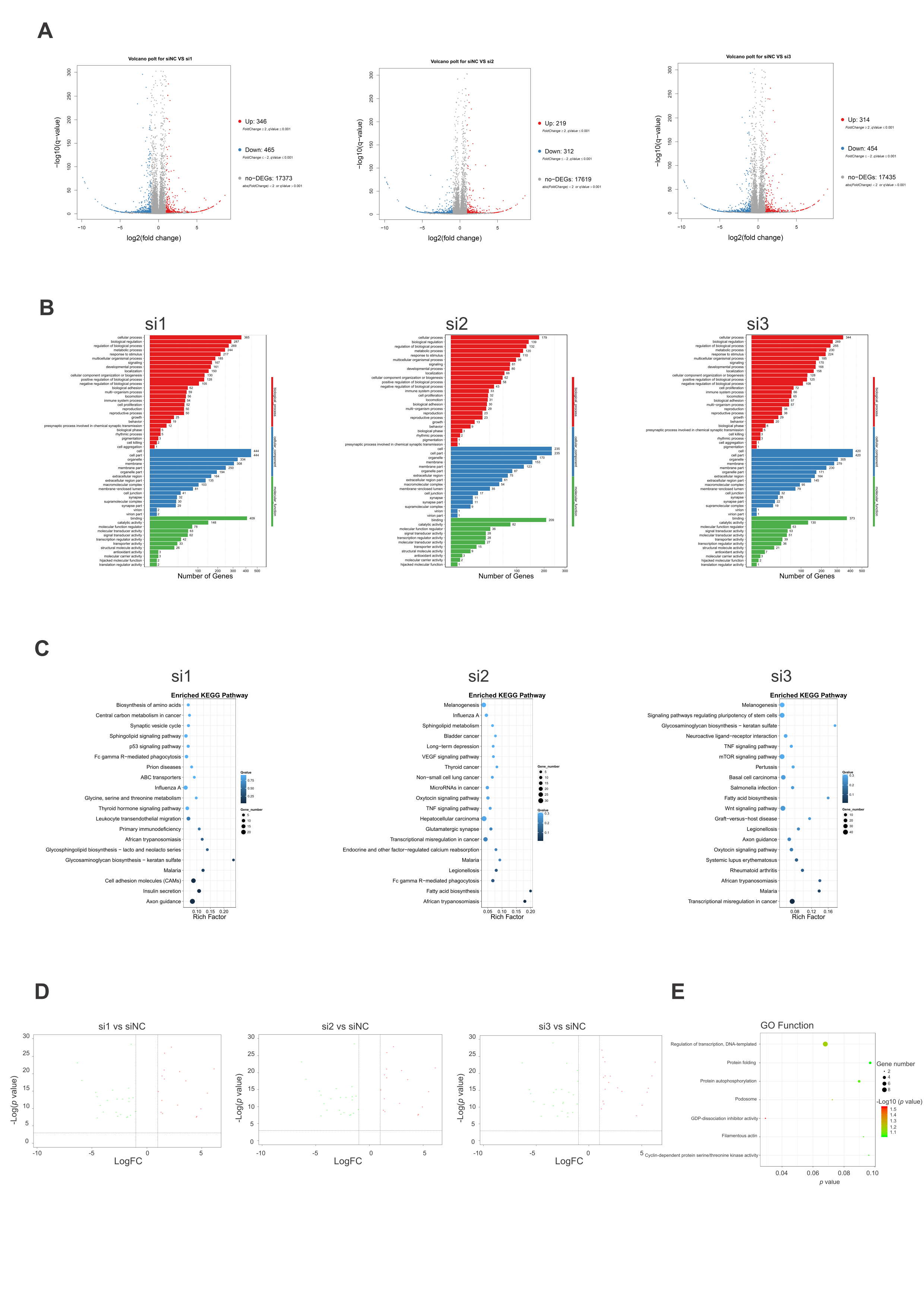

Supplement: Supplementary Figure 6 — Depletion of C1QBP downregulates VCAM-1 expression in TNBC via P65 in the nucleus. (A) Volcano plots showing the up- or downregulated transcripts in the si1, si2, and si3 groups as compared to those in siNC. Hs578T cells were transfected with siRNA to silence the expression of C1QBP, and total RNA from the groups was extracted, following which, transcriptome analyses were performed. (B) Biological processes, cellular components, and molecular functions of the GO annotations of the genes. (C) The enrichment of various mRNAs in the KEGG pathways. (D) Volcano plots of the consistently identified up- and downregulated genes. (E) GO analysis of the consistently identified up- and downregulated genes. [file Image_6.TIF]

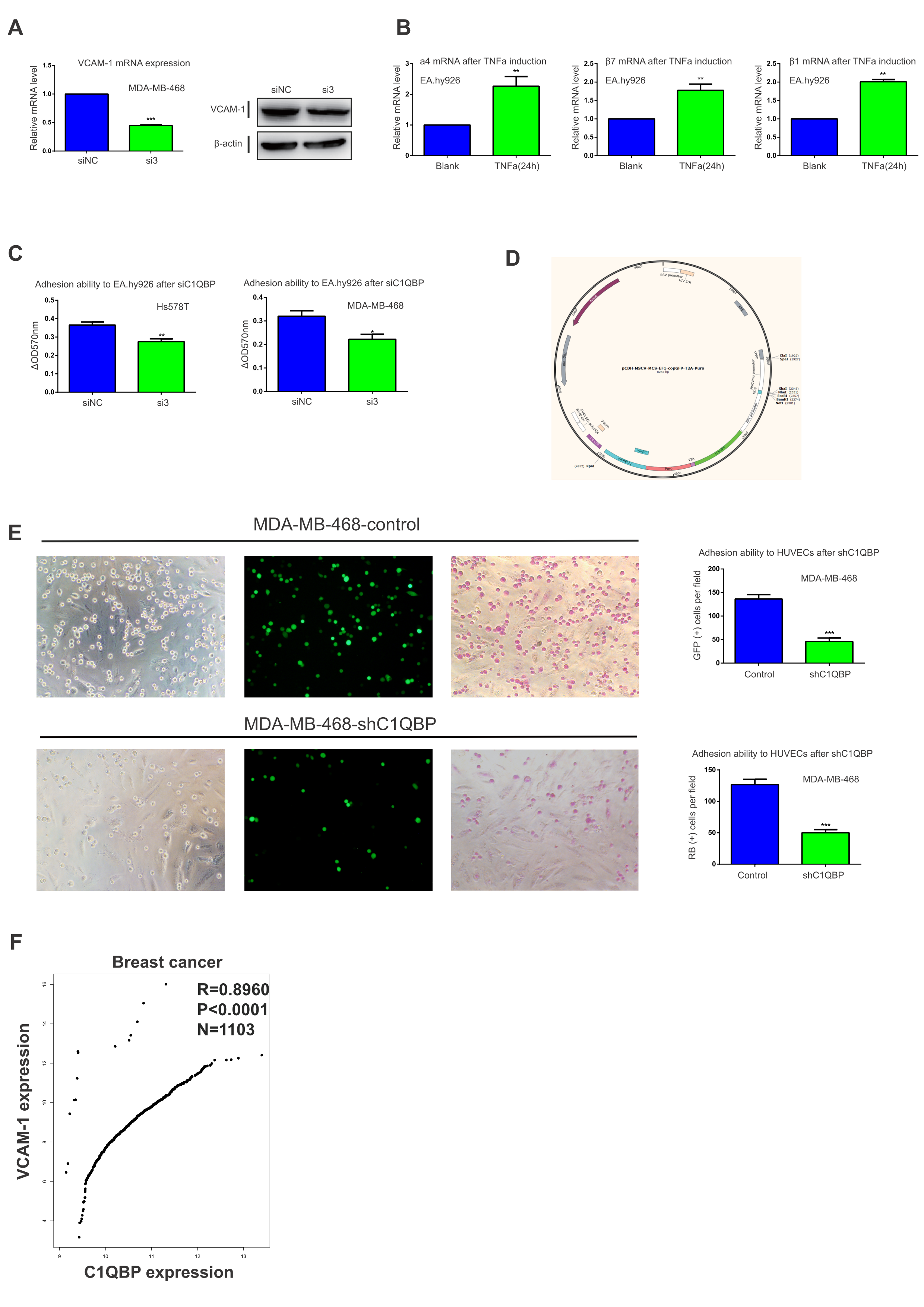

Supplement: Supplementary Figure 7 — Depletion of C1QBP downregulates VCAM-1 expression in TNBC via P65 in the nucleus. (A) Real-time qPCR and western blot were used to determine the mRNA and protein levels of VCAM-1 after transfection with si3 in the MDA-MB-468 cells for 48 h. (B) The mRNA levels of α4, β1 and β7 in EA.hy926 cells were detected upon treatment with TNFα for 24 h using real-time qPCR. (C) EA.hy926 cells were treated with TNFα (1,000 U/ml) for 24 h. Hs578T or MDA-MB-468 (transfected with siNC and si3) cells (5 × 10^4^) were incubated with EA.hy926 cells in each well. Subsequently, 0.25% RB was added to each well and the absorbance was measured at 570 nm. (D) Structure of lentiviral vectors containing luciferase. (E) HUVECs were seeded in each well of the 96-well plate (2 × 10^4^ cells per well) and treated with TNFα (1,000 U/ml) for 24 h. MDA-MB-468-control and MDA-MB-468-shC1QBP cells (5 × 10^4^ cells/200 μl) were infected with the lentiviral vectors containing GFP and added onto HUVECs. The adherent TNBC cells were visualized and photomicrographs. The number of adherent TNBC cells with green fluorescence per field was calculated. Then RB was added per well and incubated. The number of RB-stained adherent cells was also calculated. (F) Comparison of the mRNA levels of C1QBP with VCAM-1 expression in breast cancer using data from the TCGA database. Three independent experiments were used to represent the mean ± standard deviation values. *P < 0.05, **P < 0.01, and ***P < 0.0001. [file Image_7.TIF]
